# Supplementary material for: High-efficiency broadband active metasurfaces via reversible metal electrodeposition
Source: Light Sci Appl. 2026 Jan 3;15:38. doi: 10.1038/s41377-025-02136-x (PMC12764912; doi:10.1038/s41377-025-02136-x)
Supplement: Supplementary file 1 — Supplementary Materials [file 41377_2025_2136_MOESM1_ESM.pdf]

Supplementary Information for

High-efficiency broadband active metasurfaces via reversible metal electrodeposition

Li et al.

This PDF file includes:

Supplementary Text
Figures S1 to S30
Tables S1 and S2
Legends for movies S1 and S2
References

Other Supplementary Materials for this manuscript include the following:

Movies S1 and S2

Supplementary Text

1. Calculation of nominal electrodeposition thickness

The electrodeposition thickness is calculated based on the fact that the total deposit amount is related to the amount of charge used in the electrodeposition reaction. Therefore, the deposit mass m can be calculated by (*1*)

$$m = \frac{\eta_f MQ}{nF} \quad \text{(S1)}$$

where η_f is the faradic efficiency for the electrodeposition reaction at the cathode, M is the molecular weight of the deposited metal ($M = 63.55\ g \cdot mol^{-1}$ for Cu), Q is the amount of charge passed through the external circuit which can be directly read out from the potentiostat in experiments, n is the number of moles of electrons in the reaction ($n = 2$ for Cu electrodeposition), and F is Faraday constant (96485 C/ mol). The η_f is approximated to be 1, meaning that all the passed electrons contribute to the electrodeposition rather than side reactions, which is reasonable considering the extraordinary reversibility demonstrated in our experiments. Assuming that the metal atoms are densely and uniformly deposited, the thickness of the deposit layer d is given by

$$d = \frac{m}{\rho A} \quad \text{(S2)}$$

Here, ρ is the mass density, and A is the deposit area. In our experiments, the deposit area (cm^2) is much larger than the patterned area (μm^2), so we do not need to count the antenna area where there is no deposition.

2. Promising designs for easier optical switching

For our active metasurfaces with bilayer antennas, the switching mechanism relies on the electrodeposited metal to block the light from entering the cavity. Therefore, to achieve easier (or faster) switching, it would be desirable if we could use a bilayer antenna with the cavity as thin as possible. We first explored the influence of the antenna thickness on the anomalous reflection performance by simulating both the reflection and diffraction efficiencies as a function of the Au and SiO_2 antenna thicknesses (t_1 and t_2). As shown in Fig. S9, to achieve similarly good efficiencies as the pristine design ($t_1 = 40\ nm$ and $t_2 = 40\ nm$), a thicker Au layer is needed when decreasing the SiO_2 thickness. As an example, a 30 nm SiO_2 cavity paired with a 50 nm Au antenna yields similar anomalous reflection performance ($\eta_R = 73.2\%$ and $\eta_D = 89.7\%$) as that of the pristine design ($\eta_R = 76.8\%$ and $\eta_D = 90.0\%$). For this thinner-SiO_2 design, it only requires ~20 nm of Cu deposition to fully switch the beam to the specular reflection (η_D~90%) as shown in Figs. S3B and S4B.

To further improve the switching performance, we propose a design with the antennas sunken into the Au substrate. This idea originates from the fact that the first ~10 nm of Cu deposition doesn't have much influence on the anomalous reflection efficiencies, indicated by the simulations (Figs. S3 and S4) as well as the experiments (Figs. 2G and 3E). As illustrated in Figs. S3C and S4C, the sunken design achieves a considerable improvement in the required Cu thickness for switching, which is decreased to around 10 nm. Although the sunken design might lead to slightly lower reflection efficiencies for both the anomalous and specular reflection states (η_R~60%), it is still very appealing to achieve such an effective beam steering control (η_D >80%) by using just a few nanometers of Cu. This would be especially useful for applications that need fast switching speed such as in the light

detection and ranging (LiDAR) systems. We note that this performance could still be further improved if all the geometric parameters are included in the optimizations.

3. Fundamental limit of switching speed

For the RME system, the limiting factor of switching speed lies in the mass diffusion of metal ions in the electrolyte. To explore the fundamental speed limit, we can calculate the mass diffusion rate of metal ions from the electrolyte to the surface of working electrode (i.e., the metasurface). As the active metasurface requires only a little amount of metal deposition (e.g., 30 nm thick), the diffusion happens in a very small region near the electrode. Therefore, the electrode can be viewed as an infinite plane and the diffusion occurs along one dimension—normal to the plane. Such an ionic diffusion behavior can be described using the semi-infinite linear diffusion model with Cottrell equation, given by (*1*)

$$J(t) = nFC_0\sqrt{\frac{D}{\pi t}}. \quad \text{(S3)}$$

Here, $J(t)$ is the transient current density at time t, n is the number of electrons transferred per ion ($n = 2$ for Cu^{2+} ions), F is the Faraday constant, C_0 is the bulk concentration of the electroactive species (i.e., Cu^{2+} ions), D is the diffusion coefficient of the ionic species ($D = 7 \times 10^{-6}\ \mathrm{cm^2/s}$ for Cu^{2+} ions in aqueous electrolyte at 25 °C (*2*)). By integrating the Cottrell equation over time, the total charge density $Q(t)$ transferred up to time t can be obtained as:

$$Q(t) = \int_0^t J(\tau)\, d\tau = 2nFC_0\sqrt{\frac{Dt}{\pi}}. \quad \text{(S4)}$$

Subsequently, the total number of moles of transported ions $M(t)$ per unit area is expressed as:

$$M(t) = \frac{Q(t)}{nF} = 2C_0\sqrt{\frac{Dt}{\pi}}. \quad \text{(S5)}$$

For the case of 1 M Cu^{2+} electrolyte, the transported mass of Cu^{2+} is 2.11×10^{-7} mol/cm^2 within 5 ms. This, according to Eq. (S2), corresponds to a deposition of >15-nm-thick Cu. Considering that the sunken design of the metasurface only needs 10 nm Cu to achieve switching (Fig. S3), it is fundamentally possible to realize RME-based metasurfaces with a switching speed of >100 Hz, which totally fulfills the speed requirements of LiDAR (10 Hz) and video display (30 Hz) applications (*3*). It is noteworthy that some advanced antenna design which only needs an active metal volume on the level of ~5 nm^3 (*4*) might further extend the upper limit of switching speed for RME-based systems. This theoretical analysis provides promise to greatly improve the operational speed of RME-based metasurfaces, which can be achieved via a hybrid engineering on the metasurface design and the electrochemical system (e.g., using ionic liquid electrolyte to enable higher overpotential deposition with faster nucleation speed). As a preliminary experimental demonstration, Figure S16 clearly shows that the switching speed substantially increases with enlarged applied voltage magnitude and a 100 ms switching, both for deposition and stripping, can be readily achieved.

4. Considerations of selecting Cu as working metal

In this work, the selection of Cu as the working metal in RME operations was primarily based on electrochemical considerations. Among various metals, the moderate reduction potential of Cu, +0.34 V vs. standard hydrogen electrode (SHE), indicates its relative ease of both electrochemical deposition and stripping, which is crucial for RME operation. To date, the RME-Cu system has demonstrated remarkable capability as a highly stable, durable, and fast-switching active material platform for broad applications (*5–7*). For example, up to 10000 stable RME cycles have been readily achieved in Cu-based electrochromic systems (*7*). The excellent electrochemical robustness of Cu/Cu^{2+} makes it

highly appealing for demonstrating the RME-based dynamic metasurfaces with high performance.

Indeed, there are other metals available for RME operations, such as silver (Ag), aluminum (Al), and lithium (Li), which feature higher reflectivity than Cu in shorter visible wavelengths. However, their (electro)chemical properties mainly made them less ideal, especially for manipulating light-matter interactions at subwavelength scales which demands high-quality electrodeposition (e.g., uniform and dense). For example, Al (-1.68 V vs. SHE) is highly active and is much more difficult to reduce than Cu, rendering the RME operation of Al rather challenging. Such systems demand air- or water-free working environments and carefully tailored electrolytes to suppress side reactions. Li is even more active than Al with highly negative reduction potential (-3.04 V vs. SHE), posing additional complexities to the device encapsulation and optical characterization. In addition, when Li is formed, it reacts with the electrolyte form a chemically complex and heterogeneous layer, the so-called "solid-electrolyte interphase (SEI)", whose optical constants are challenging to characterize accurately. Li can also form alloys with Au (*8*), making it difficult, if not impossible, to predict the nanoantennas' permittivity over prolonged cycles. As for Ag, it is quite difficult to achieve highly reversible and stable RME switching like RME of Cu, according to both our own experiences (*6*, *9*) and literature demonstrations (*7*, *10*). This may be attributed to the inertness of Ag (+0.80 V vs. SHE), which makes it more difficult to electrochemically strip compared to Cu. Additionally, the self-dissolution issue in typical RME-Ag systems results in undesirable instability (*6*). While the use of Ag in RME systems is technically challenging, it remains an important and promising candidate for active metasurfaces operating at shorter visible wavelengths, which requires a dedicated study of electrochemical and optical behavior at the microscopic level.

It is worth noting that RME of Cu can achieve beam-steering performance comparable to that of other metals in the red-visible and near-IR spectral ranges (e.g., 685 nm), as supported by our simulation results in Fig. S17. The experimental measurements of >90% diffraction efficiency and >10 dB signal-to-noise ratios (SNR) in both the red-visible and near-infrared (IR) ranges have been sufficient to showcase the great potential of RME-Cu for dynamic metasurfaces.

5. Influence of deposition surface morphology on beam-steering performance

For the RME-based dynamic metasurfaces, a uniform and smooth surface morphology of electrodeposited Cu is essential for achieving high-performance beam steering. To quantitatively assess the impact of surface morphology on beam-steering behavior, we carried out a series of simulations. First, from the perspective of phase and reflectivity modulation (Fig. S18), we found that the reflection phase remained nearly constant (~150°) across different positions along the trapezoidal antenna when the deposited Cu layer is thicker than 30 nm. This suggests that, even with substantial thickness variation, a desirable reflection wavefront can still be achieved as long as the deposited Cu layer is sufficiently thick. This is verified by further simulations of the far-field reflection intensity profiles. These simulations involved two representative scenarios: (i) thickness variation across different antenna units (Fig. S19), and (ii) variation within a single antenna (Fig. S20). For each average Cu thickness (30, 40, and 50nm), ten random sets of Cu thickness values were generated using a Gaussian distribution with standard deviations of 5%, 10%, 15%, and 20%.

While increasing the deposited metal thickness helps preserve reliable phase modulation, our simulation results also highlight the importance of film uniformity at small deposition thickness. Significant non-uniformity may lead to large degradation and fluctuation in reflection signal strength

and beam quality. These findings underscore that achieving stable, high-SNR beam steering in dynamic metasurfaces requires precise control of subwavelength light–matter interactions. In our experiments, the observed high diffraction efficiencies and SNRs are attributed to the highly uniform and reversible Cu electrodeposition, as evidenced by the top-view SEM images in Fig. S21. To further support this, we characterized the surface morphology of the electrodeposited Cu using atomic force microscopy (AFM), as shown in Fig. S22. The results show that, for a nominal deposition thickness of 30 nm, the electrodeposited film exhibits a root mean square roughness (Rq) of 2.31 nm and an average roughness (Ra) of 1.85 nm, comparable to that of Cu deposited by e-beam evaporation. These results confirm the high-quality and uniform deposition of Cu on Au electrodes.

Here we would like to provide more details on why Cu electrodeposition on Au substrates can achieve such excellent uniformity and surface roughness in a reversible and stable manner. First, the high electrical conductivity of Au substantially reduces the potential drop (ohmic drop) during electrodeposition, ensuring uniform current density distribution and consistent deposition thickness. In addition, Au offers a favorable lattice match with Cu, as both metals have face-centered cubic (FCC) crystal structures with similar lattice parameters (0.41 nm for Au and 0.36 nm for Cu). Therefore, when Cu atoms are deposited on Au surface, they can more readily conform to the crystallographic orientation of Au, suppressing random nucleation, limiting defects, and favorably forming a dense and smooth thin film. Furthermore, the great electrochemical inertness of Au makes it highly stable during RME operations, limiting side reactions and ensuring deposition qualities. These combined electrochemical benefits make it possible to realize highly switchable and stable dynamic metasurfaces with extraordinary optical performance, as demonstrated in this work.

6. Prospect of achieving RME-based metasurfaces working in transmission mode

Dynamic metasurfaces operating in transmission mode can offer broad opportunities for practical applications. To date, many previous studies on electrochromic devices, such as smart windows and radiative thermoregulators, have demonstrated extraordinary optical tunability by performing RME on transparent conductive electrodes (*5*, *9*). Building upon these electrochemical techniques and integrating them with proper nanophotonic designs, it is theoretically feasible to develop dynamic transmission metasufaces via RME with high optical performance.

That said, experimentally realizing RME-based metasurfaces that operate in transmission mode with desirable optical tunability remains nontrivial. Key challenges include the limitation of electrode conductivity and the issue of lattice mismatch between the electrode and the deposited metal. These factors can hinder the uniformity of electrodeposited metal and degrade optical responses in nanostructured metasurfaces. For example, Cu electrodeposition on indium tin oxide (ITO) or graphene often leads to the formation of isolated particles rather than continuous films (Fig. S23A). To mitigate this, one effective strategy is to introduce platinum (Pt) nanoparticles or ultrathin Pt films as seed layers, which enhance nucleation while preserving visible transparency (*6*). As a preliminary demonstration, we used e-beam evaporation to deposit a 2 nm Pt layer on the commercial ITO glass and achieved a continuous Cu film via electrodeposition (Fig. S23B). Another approach to improve deposition quality is to add polymer inhibitors into the electrolyte to promote more uniform metal growth. We are optimistic that, with more researchers' efforts in this direction, high-performance RME-based metasurfaces operating in transmission mode can also be realized in the near future.

7. Influence of potential laser heating on beam-steering performance

Under prolonged high-intensity laser illumination, light absorption by the metasurface, electrode, or electrolyte can increase the device temperature, in the absence of an active cooling system. This temperature change may affect both the thermodynamic and kinetic aspects of the electrochemical system. Generally, elevated temperatures accelerate electrochemical reactions by lowering activation barriers and increasing ion mobility. As stated in a classic electrochemistry textbook (*1*), "A general rule of thumb is that the reaction rate doubles for each 10 °C increase in temperature."

From a thermodynamic perspective, higher temperatures provide more thermal energy to overcome the energy barrier required for nucleation, thereby speeding up RME operations. Specifically, the relationship between nucleation rate and temperature is given by (*1*):

$$J_0(T) = A_0 \exp(-\frac{\Delta G_{\text{crit}}}{kT}) \quad \text{(S6)}$$

where $J_0(T)$ is the nucleation rate (in cm^{-2} s^{-1}), A_0 is a constant, ΔG_{crit} is the Gibbs free energy for critical cluster formation, and k is the Boltzmann's constant. Equation (S6) indicates that the nucleation process is strongly temperature dependent.

In terms of kinetics, temperature can also greatly influence the ion diffusivity. According to Nernst-Einstein relationship, the ion diffusivity in dilute solutions is directly proportional to temperature (*1*):

$$D_i = RTu_i \quad \text{(S7)}$$

where D_i is the diffusivity of species i, R is the ideal gas constant, and u_i is the mobility of the ion. In light of Eqs. (S6) and (S7), it is crucial to consider temperature effects for the practical implementation of electrochemical devices. Possible mitigation strategies include thermal

management (e.g., active cooling systems) or adaptive control algorithms that dynamically tune the applied voltage and duration in response to real-time laser intensity or device temperature.

We also note that, while optical heating may raise concerns about system stability, it can simultaneously enhance the switching speed of the device. To explore this, we conducted a series of experiments to evaluate the influence of temperature on switching speed. As shown in Fig. S24, the results reveal a clear increase in switching speed under elevated temperatures, in line with the theoretical expectations.

8. Possibility of achieving continuous-angle beam steering

To provide a prospect of our RME-based metasurface platform, we have conducted a series of simulations and experiments that reveal its promise in realizing continuous-angle beam steering. Achieving such functionality requires actively tailoring the meta-atom periodicity and locally modulating the reflection phase across a full 0 to 2π range, to enable a spatially uniform phase gradient. To address this, we propose a modified design based on grating-shaped MIM resonators (Fig. S25A). The grating structure makes it possible to individually control each unit-cell element and modulate the corresponding local phase response by the applied voltage. The local phase modulation is enabled by laterally depositing metal on the sidewall of the Au gratings, which effectively changes their widths. As demonstrated in Fig. S25B, the lateral control of Cu deposition achieves a desirable near-2π phase coverage, laying the foundation of beam steering with continuous angle modulation. Therefore, by individually controlling the applied voltages at different gratings, various super cell patterns can be achieved with different phase gradient, leading to continuously tunable steering angles of the reflected beam (Fig. S25C). As a preliminary experimental demonstration, Figure S25D shows the nanorod

width effectively increases after electrodeposition, validating the feasibility of lateral RME control. These results illustrate the potential of our RME platform to enable more light-manipulation functionalities.

9. Practical implementation of RME-based metasurfaces

It is worth noting that electrochemical devices utilizing liquid electrolytes have already been widely adopted in many commercial systems, including lithium-ion batteries and electrochromic windows. In fact, lithium-ion batteries predominantly use liquid electrolytes, which have been essential in smartphones, laptops, and electric vehicles. These examples illustrate that liquid electrolyte-based systems are not inherently incompatible with scalable technologies, and similar engineering strategies can be extended to electrochemical photonic platforms.

To provide a proof-of-concept demonstration of our electrochemical systems, we fabricated a sealed electrochemical cell using waterproof tape and epoxy glue, as shown in Fig. S26. This device exhibited over 100 stable cycles with >90% diffraction efficiencies, consistent with the in-situ and ex-situ results presented in the main text (Fig. 4). We believe that the advanced packaging and encapsulation technologies developed for the battery industry could greatly facilitate the transition of electrochemical devices, including ours, toward practical applications.

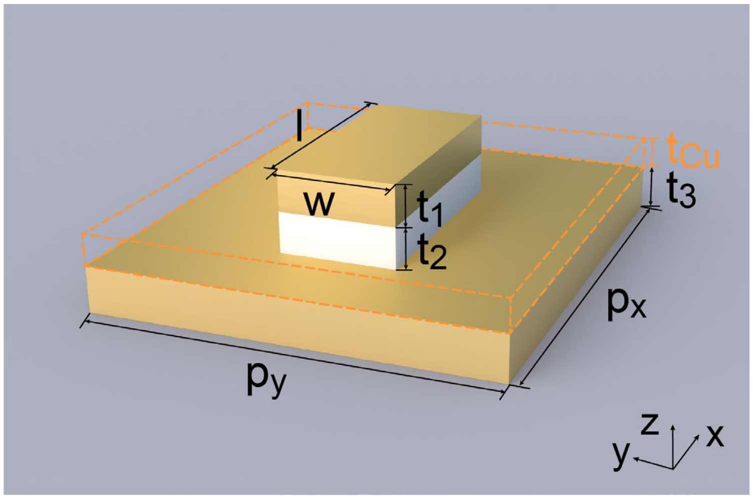

Figure S1. Simulation model of tunable metal-insulator-metal resonator. For the visible wavelength ($\lambda = 600\,nm$), $P_x = P_y = 260\,nm$, $l = 200\,nm$, $w = 70\,nm$, $t_1 = t_2 = 40\,nm$, $t_3 = 100\,nm$, $t_{Cu} = 30\,nm$. For the near-IR wavelength ($\lambda = 1500\,nm$), $P_x = P_y = 520\,nm$, $l = 420\,nm$, $w = 300\,nm$, $t_1 = t_2 = 50\,nm$, $t_3 = 100\,nm$, $t_{Cu} = 50\,nm$. For the mid-IR wavelength ($\lambda = 5000\,nm$), $P_x = P_y = 2000\,nm$, $l = 1600\,nm$, $w = 1400\,nm$, $t_1 = t_2 = 100\,nm$, $t_3 = 100\,nm$, $t_{Cu} = 100\,nm$. The light is normally incident with the polarization along the y-axis.

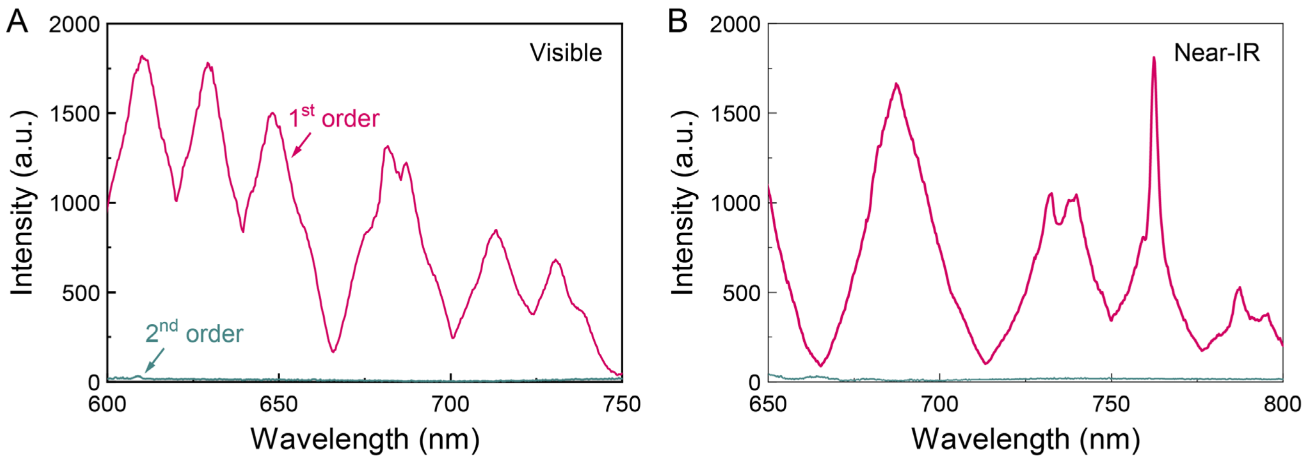

Figure S2. Measured intensity of 1st and 2nd orders of anomalous reflection for samples designed for visible (A) and near-IR (B) regimes at the stripped state. Data was obtained via the angle-resolved spectroscopy system. The multiple peaks result from combining spectra measured at different angles, with each angle exhibiting a dominant wavelength. The results show that the higher order intensity is negligibly small compared to the dominant orders.

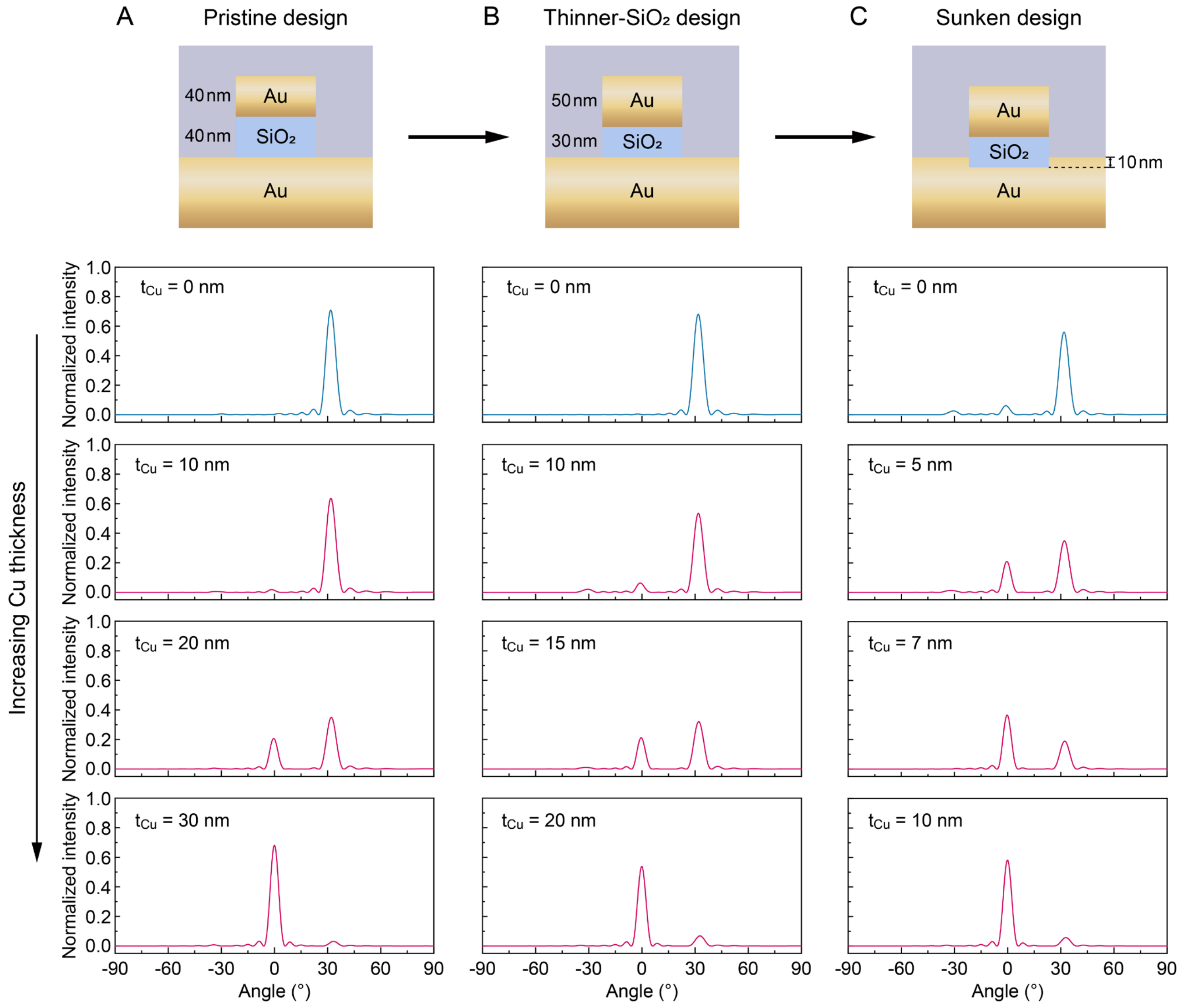

Figure S3. Simulated far-field intensity profiles with increasing Cu deposition thickness for the pristine (A), thinner-SiO_2 (B), and sunken (C) designs. The results are normalized to the peak reflection intensity of an Ag mirror. The geometric parameters not indicated in the schematics are identical to those in Fig. 3 in the main text. The light is normally incident with a wavelength of 685 nm.

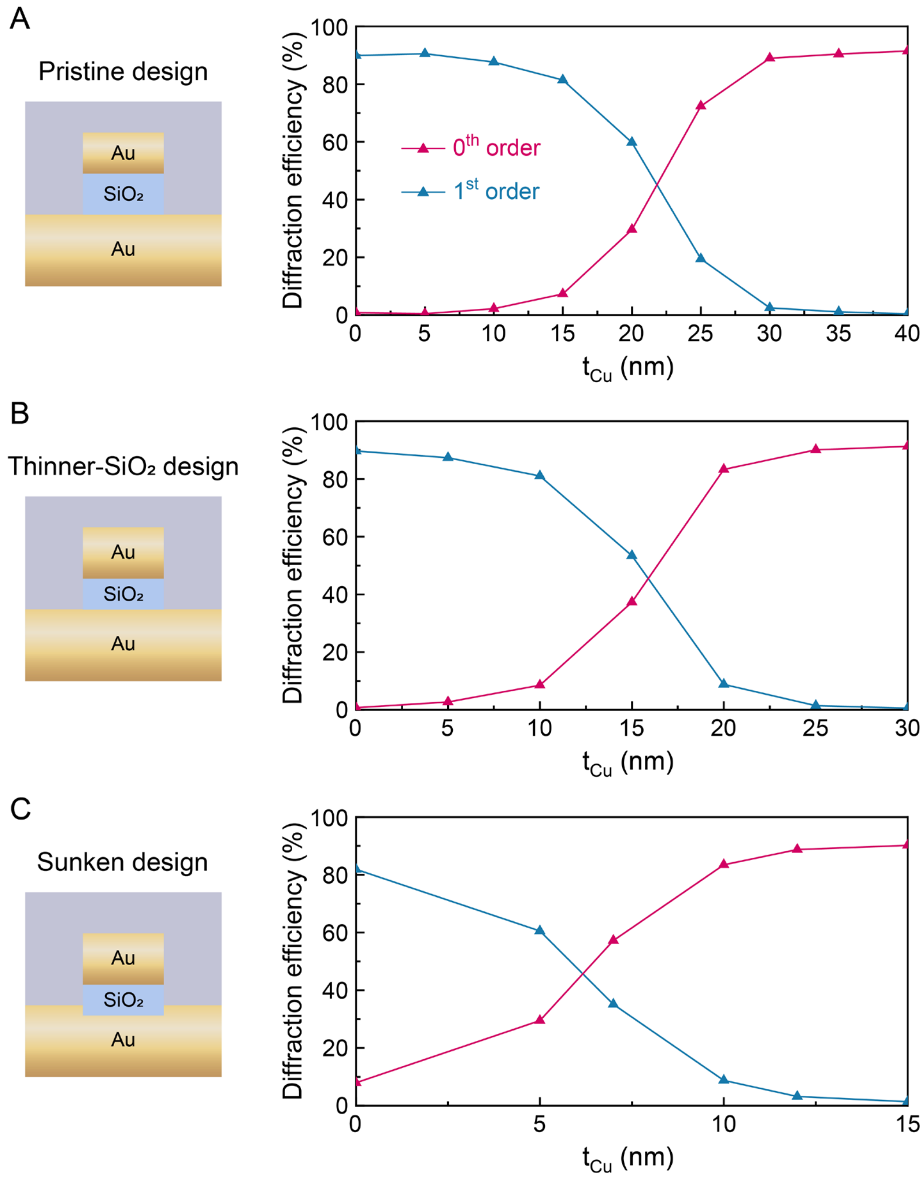

Figure S4. Simulated diffraction efficiencies with increasing Cu deposition thickness for the pristine (A), thinner-SiO_2 (B), and sunken (C) designs. The geometric parameters in the simulations are the same as in Fig. S3.

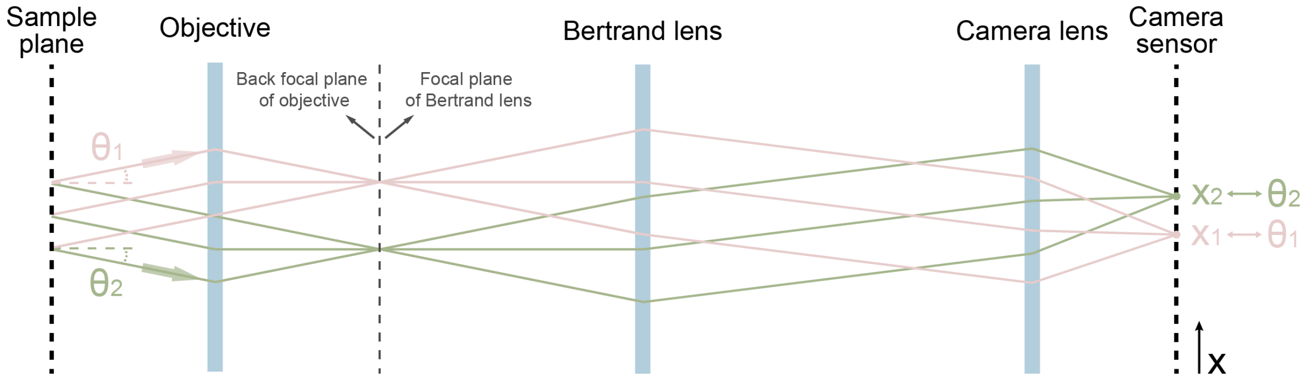

Figure S5. Schematic of Fourier plane imaging in the microscope with Bertrand lens. This technique is also known as back focal plane imaging as the Bertrand lens is essentially imaging the back focal plane of the objective (*11*). As illustrated in the schematic, the reflected light at different angles (θ_1 and θ_2) from the sample is focused onto different positions on the camera sensor (x_1 and x_2).

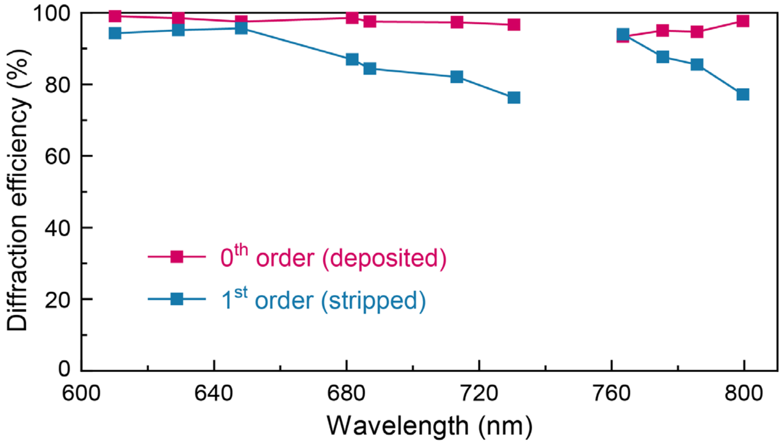

Figure S6. Measured diffraction efficiencies for the metasurfaces operating in the visible and near-IR regimes.

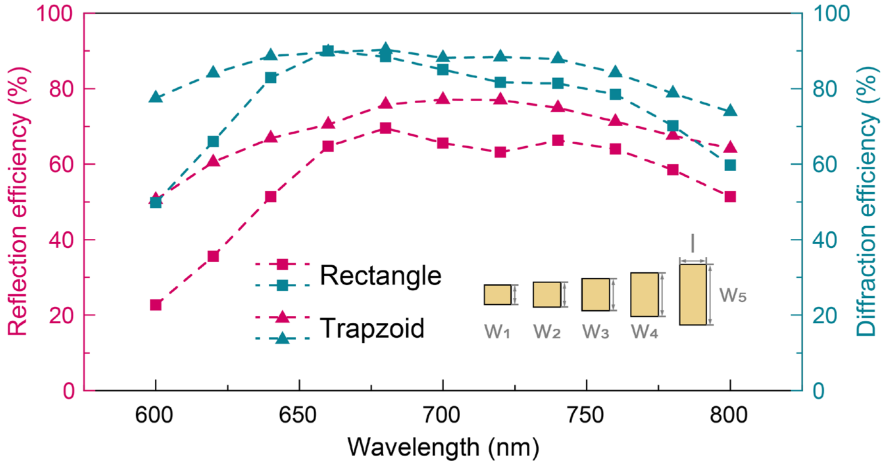

Figure S7. Comparison of simulated anomalous reflection efficiencies between trapezoidal- and rectangular-antennas metasurfaces at different wavelengths. In the simulation, the trapezoidal-antenna metasurface uses the same geometric parameters as in Fig. 3 in the main text. For the rectangle-antenna metasurface (inset shows the supercell top view), the parameters are $P_x = 1300\ nm$, $P_y = 220\ nm$, $l = 180\ nm$, $w_1 = 30\ nm$, $w_2 = 80\ nm$, $w_3 = 95\ nm$, $w_4 = 120\ nm$, $w_5 = 170\ nm$, $t_1 = 40\ nm$, $t_2 = 40\ nm$, $t_3 = 100\ nm$. The light is normally incident with the polarization along the w direction.

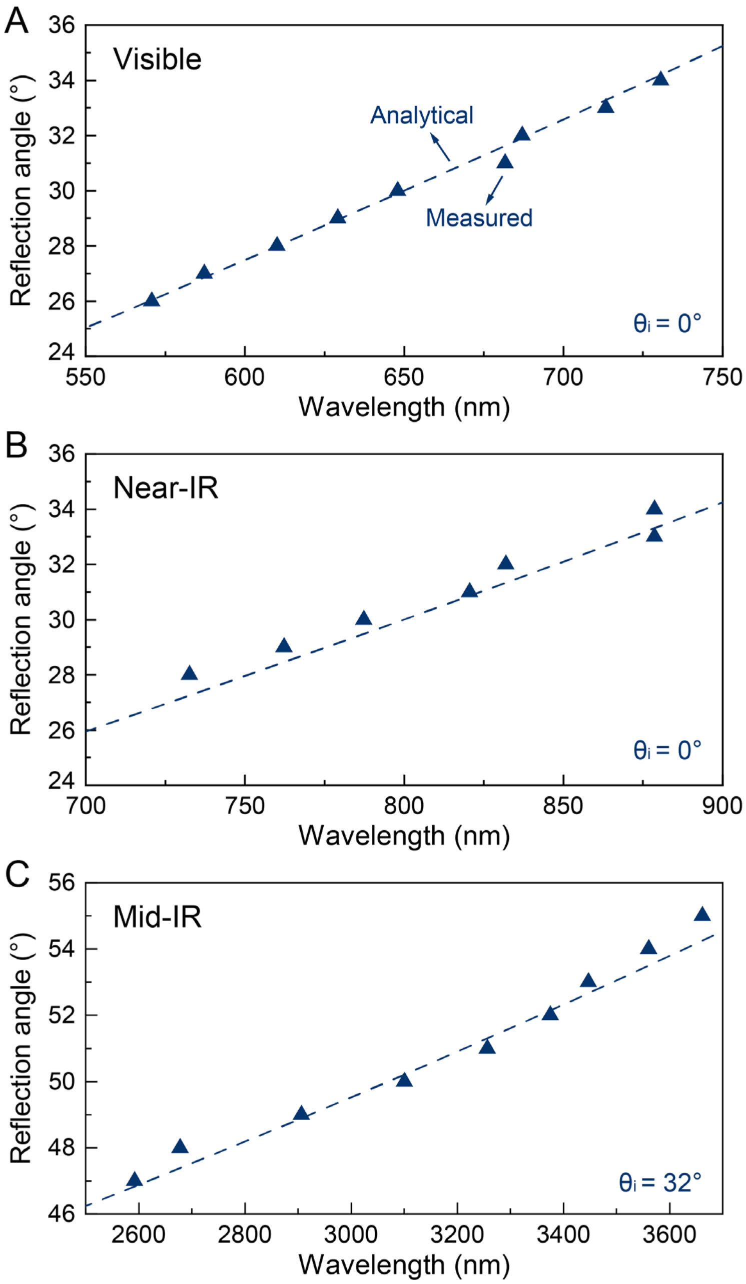

Figure S8. Comparison of anomalous reflection angles between the analytical and the measured results for the metasurfaces operating in the visible (A), near-IR (B), and mid-IR (C) regimes. The analytical results are given by the generalized Snell's law, $\theta_{\mathrm{r}} = \arcsin[\sin(\theta_{\mathrm{i}}) + \lambda/P_x]$.

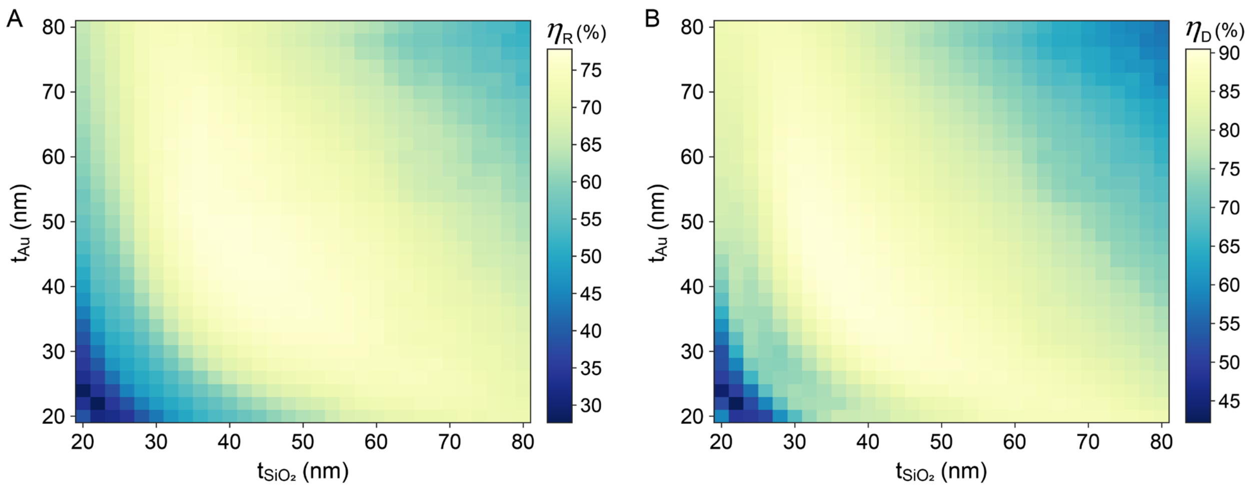

Figure S9. Anomalous reflection (A) and diffraction (B) efficiencies as a function of Au and SiO_2 antenna layer thicknesses for the sample operating in the visible regime. The other geometric parameters are the same as in Fig. 3 in the main text. The light is normally incident with a wavelength of 685 nm.

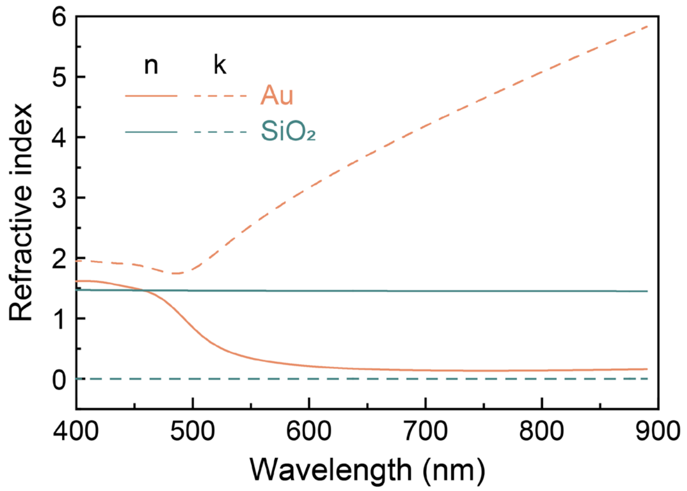

Figure S10. Measured optical properties of electron-beam evaporated Au and SiO_2 at visible and near-IR wavelengths.

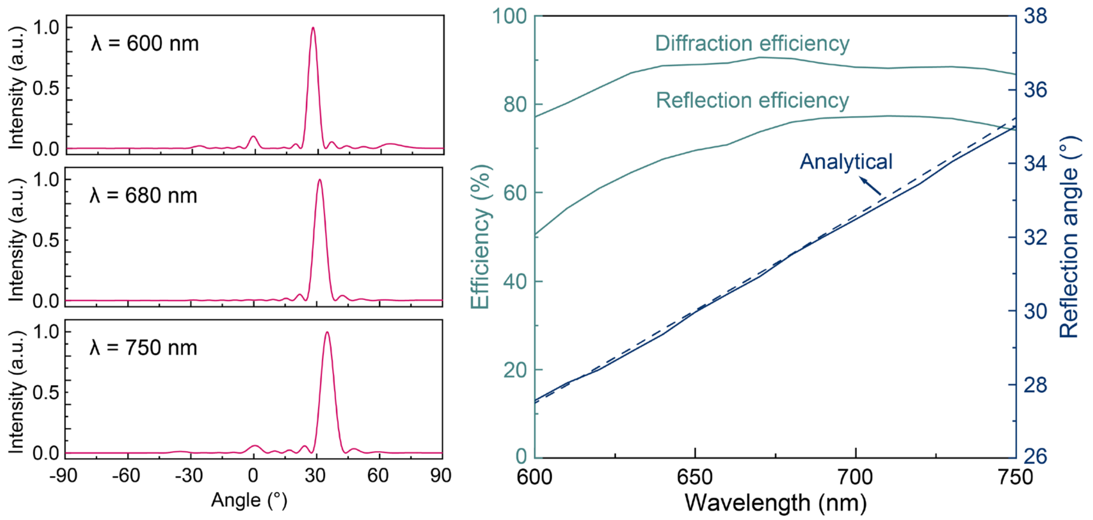

Figure S11. Simulated anomalous reflection of the metasurface designed for the visible regime (stripped state). For the simulation model, the geometric parameters are the same as in Fig. 3 in the main text. The optical properties of Au and SiO_2 are plotted in Fig. S10. The dashed line is the analytical prediction of the reflection angles based on the generalized Snell's law, which is given by $\theta_{\mathrm{r}} = \arcsin(\lambda/P_x)$ for the case of normally incident light.

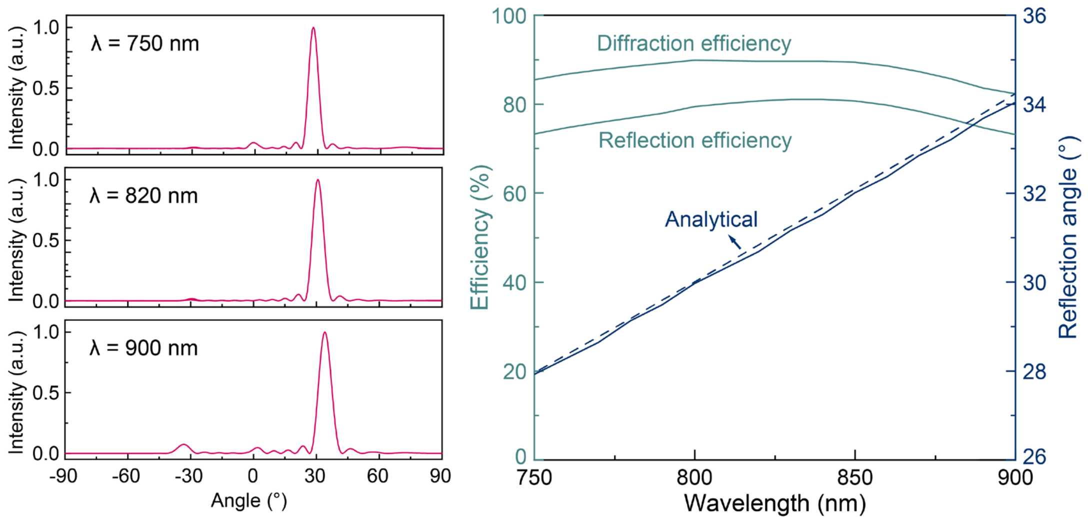

Figure S12. Simulated anomalous reflection of the metasurface designed for the near-IR regime (stripped state). For the simulation model, the geometric parameters are $P_x = 1600$ nm, $P_y = 270$ nm, $l = 1150$ nm, $w_l = 185$ nm, $w_s = 95$ nm, $t_1 = 45$ nm, $t_2 = 40$ nm, $t_3 = 100$ nm. The optical properties of Au and SiO_2 are plotted in Fig. S10. The dashed line is the analytical prediction of the reflection angles based on the generalized Snell's law, which is given by $\theta_\mathrm{r} = \arcsin(\lambda/P_x)$ for the case of normally incident light.

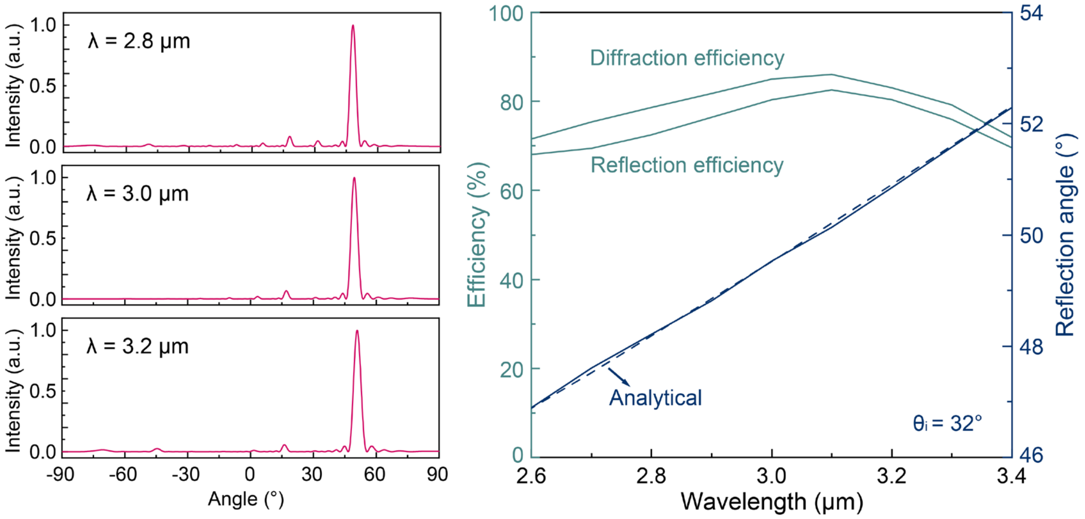

Figure S13. Simulated anomalous reflection of the metasurface designed for the mid-IR regime (stripped state). For the simulation model, the geometric parameters are $P_x = 13$ μm, $P_y = 1950$ nm, $l = 12.15$ μm, $w_l = 1350$ nm, $w_s = 400$ nm, $t_1 = 80$ nm, $t_2 = 360$ nm, $t_3 = 100$ nm. The optical properties of Au and SiO_2 are adopted from literature (*12*, *13*). The dashed line is the analytical prediction of the reflection angles based on the generalized Snell's law, which is given by $\theta_{\mathrm{r}} = \arcsin[\sin(\theta_{\mathrm{i}}) + \lambda/P_x]$ with an incident angle θ_{i} of 32°.

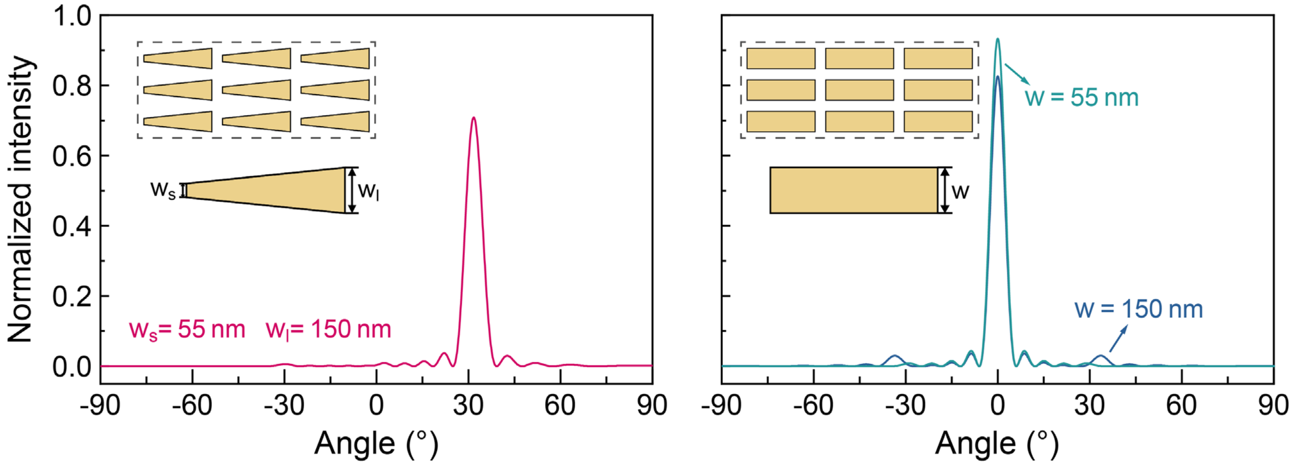

Figure S14. Comparison of simulated reflection profiles between trapezoidal- and rectangular-antenna metasurfaces. The simulation parameters not indicated in the schematics are identical to those in Fig. 3 in the main text. The polarization of incident light is along the width (*w*) direction. The results show that the anomalous reflection observed in the trapezoidal antennas originates from the phase gradient introduced by the width variation, rather than purely from periodicity as in diffractive gratings.

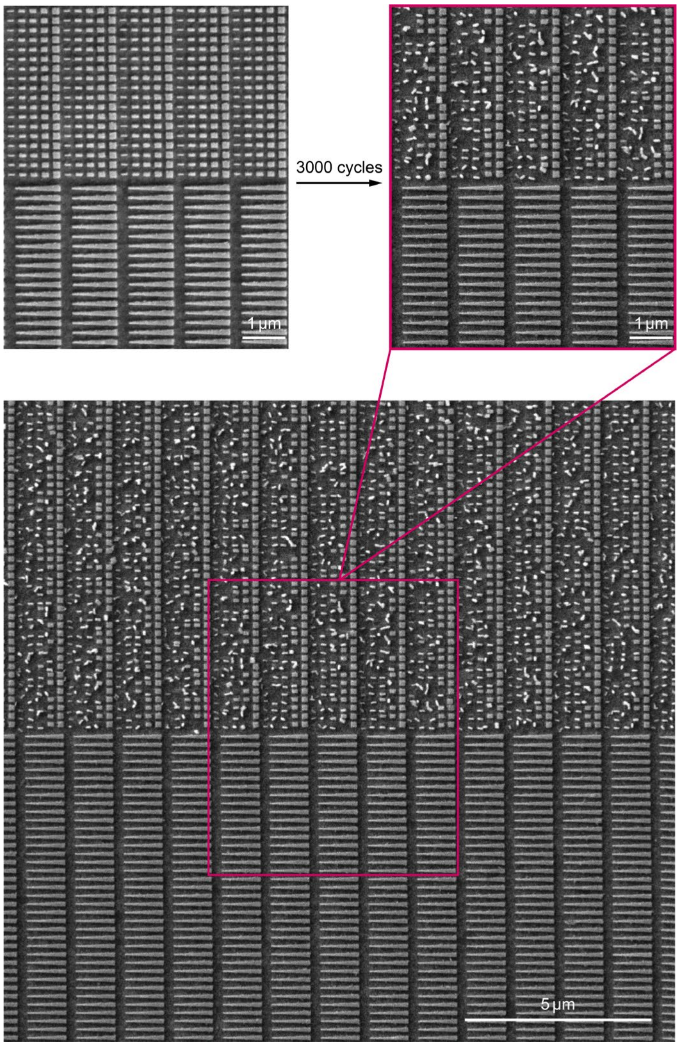

Figure S15. Comparison of cyclability between trapezoidal- and rectangular-antenna gradient metasurfaces. Two arrays were fabricated side by side to ensure identical fabrication and electrochemical conditions. The cycling test was conducted under the same conditions as those in Fig. 4E of the main text. The results indicate that the trapezoidal design exhibits better adhesion to the substrate than the discrete-nanorod configuration due to a larger contact area, which is important to the device durability. Importantly, no signs of incomplete stripping or residual Cu were observed after 3000 cycles, underscoring the robust cycling performance of our RME-based metasurfaces.

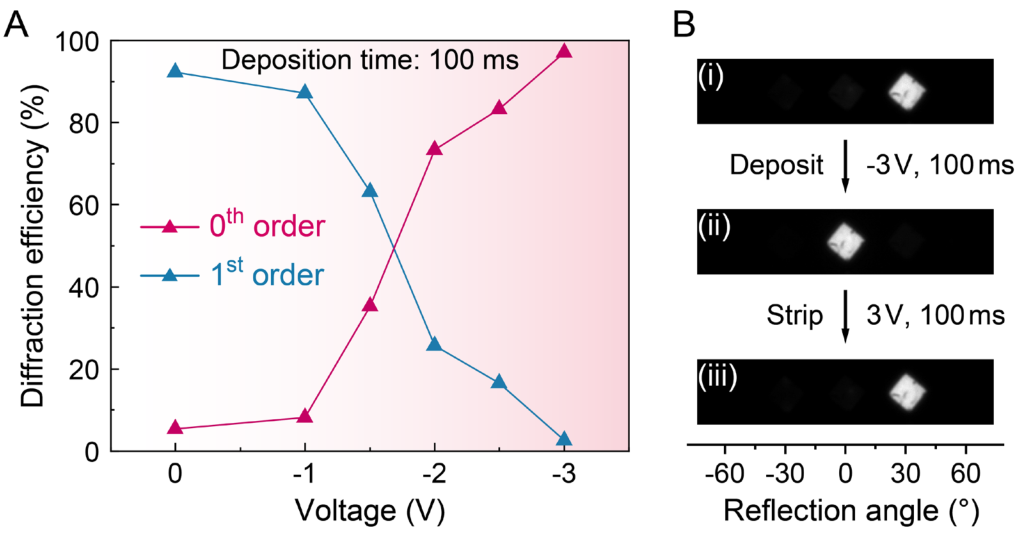

Figure S16. Realization of 100 ms switching speed by increasing the applied voltage in our RME-based metasurface. (A) Measured diffraction efficiencies after electrodepositing Cu for 100 ms, as a function of applied voltage. (B) Microscope Fourier imaging of the reflected beam profiles for the metasurface as fabricated (i), after electrodepositing Cu at -3 V (ii), and after stripping Cu at 3 V (iii) with a switching time slot of 100 ms.

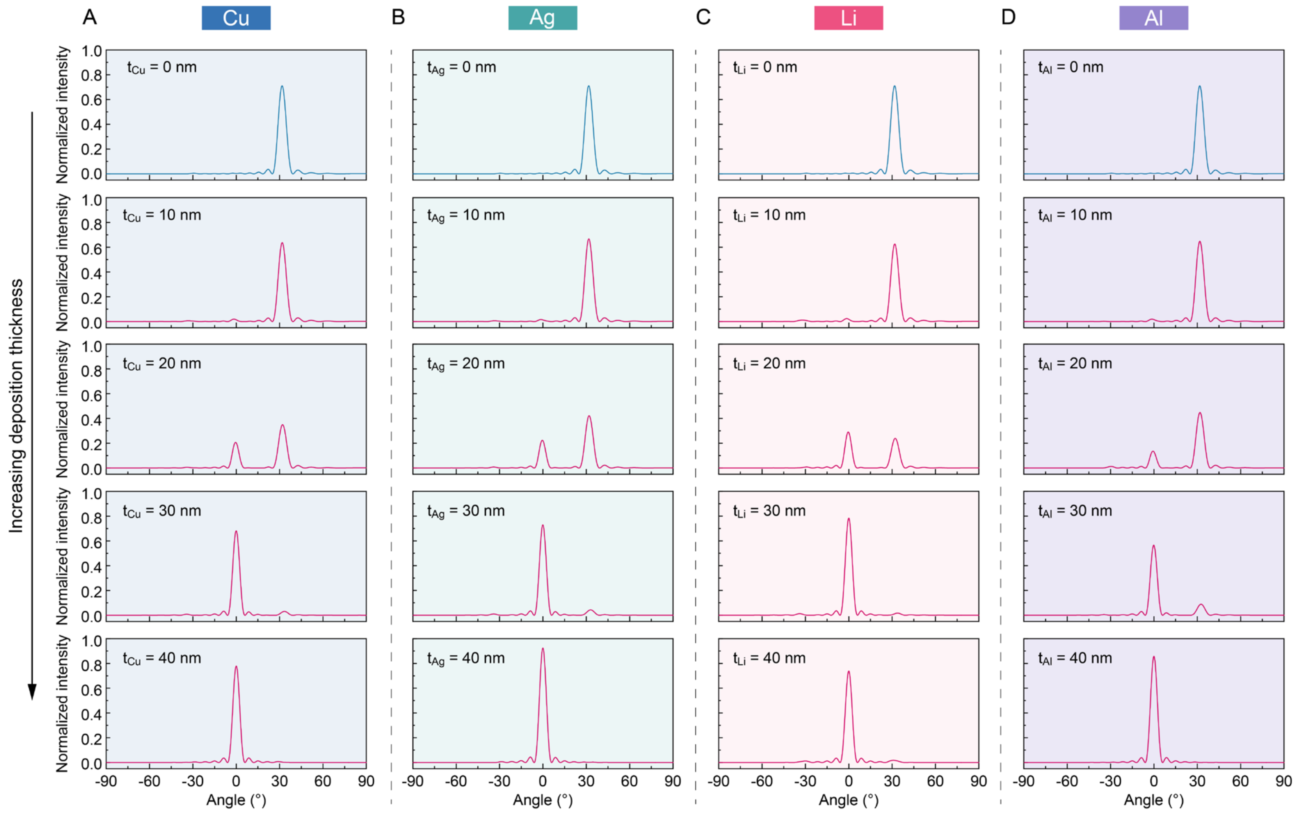

Figure S17. Simulated far-field intensity profiles during electrodeposition for RME operations using Cu (A), Ag (B), Li (C), and Al (D). In the simulations, parameters not indicated in the annotations are identical to those in Fig. 3 of the main text, and the optical properties of Cu (*14*), Ag (*15*), Li (*16*), and Al (*17*) are obtained from the literature. We note that, while various metals theoretically exhibit good beam-steering ability, the successful uniform and dense deposition of Cu does not necessarily translate to other metal systems, which might lead to significantly poorer performance in experiments.

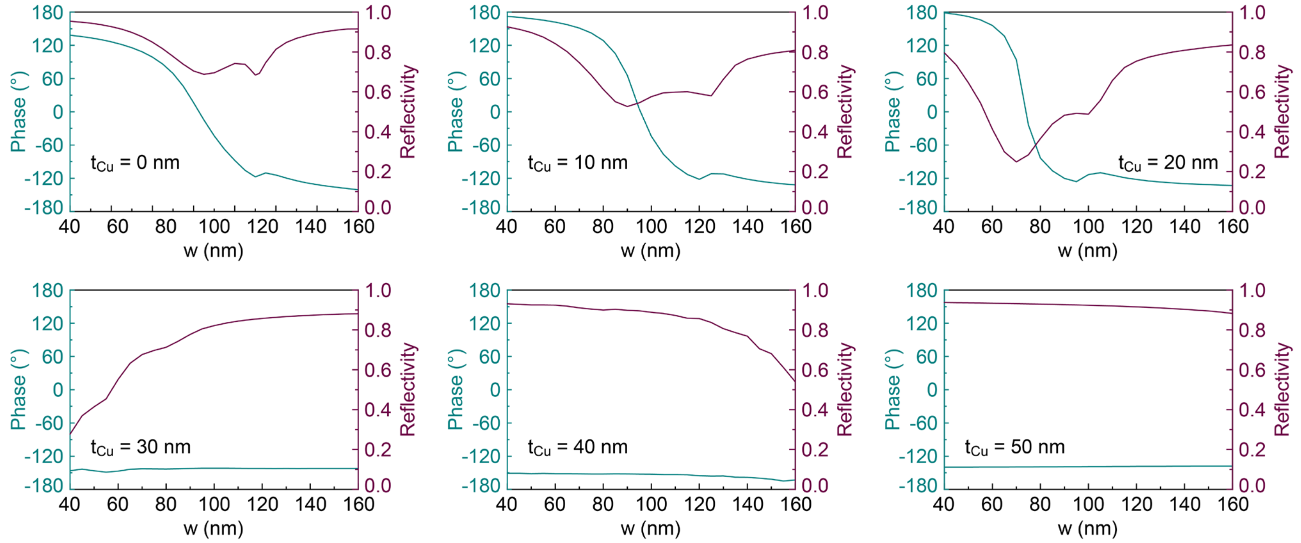

Figure S18. Simulated phase and reflectivity profiles for a rectangular antenna as a function of width (w), for different Cu deposition thicknesses (t_{Cu} = 0-50 nm). Simulation parameters not explicitly stated are identical to those used in Fig. 3C of the main text. The panels for $t_{Cu} = 0$ nm and $t_{Cu} = 30$ nm are reproduced from Fig. 3C of the main text for comparison.

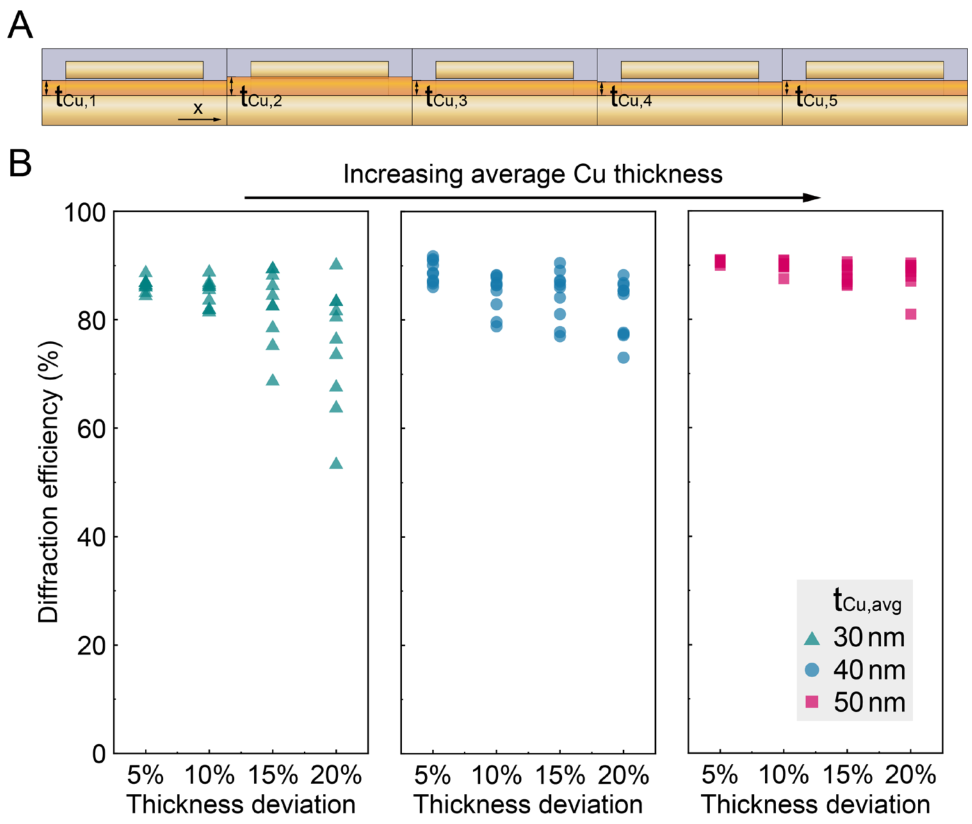

Figure S19. Influence of deposition thickness variation across different antennas on the beam-steering performance. (A) Schematic illustration of Cu thickness variation among five antenna units. (B) Simulated diffraction efficiency of beam-steering metasurfaces under various levels of Cu thickness deviation. For each average Cu thickness ($t_{\mathrm{Cu,avg}}$ = 30, 40, or 50 nm), ten random sets of Cu thicknesses ($t_{\mathrm{Cu,1}}$ to $t_{\mathrm{Cu,5}}$) were generated based on a Gaussian distribution with standard deviations of 5%, 10%, 15%, or 20%, representing thickness variation across antennas. Simulation parameters not explicitly stated are identical to those used in Fig. 3 of the main text.

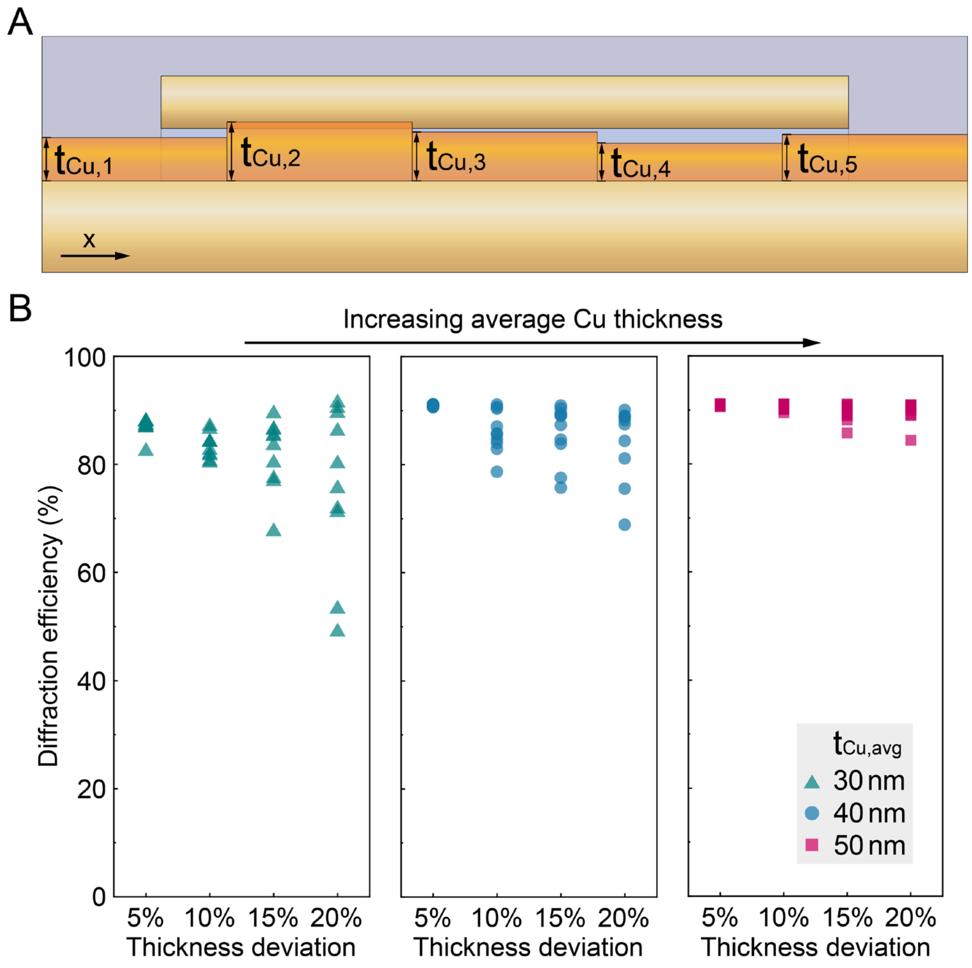

Figure S20. Influence of deposition thickness variation within individual antenna on the beam-steering performance. (A) Schematic illustration of Cu thickness variation across five segments within a single antenna unit. (B) Simulated diffraction efficiency of beam-steering metasurfaces under different levels of intra-antenna Cu thickness deviation. The segmental thickness values were generated using the same statistical settings as in Fig. S19. Simulation parameters not explicitly stated are identical to those used in Fig. 3 of the main text.

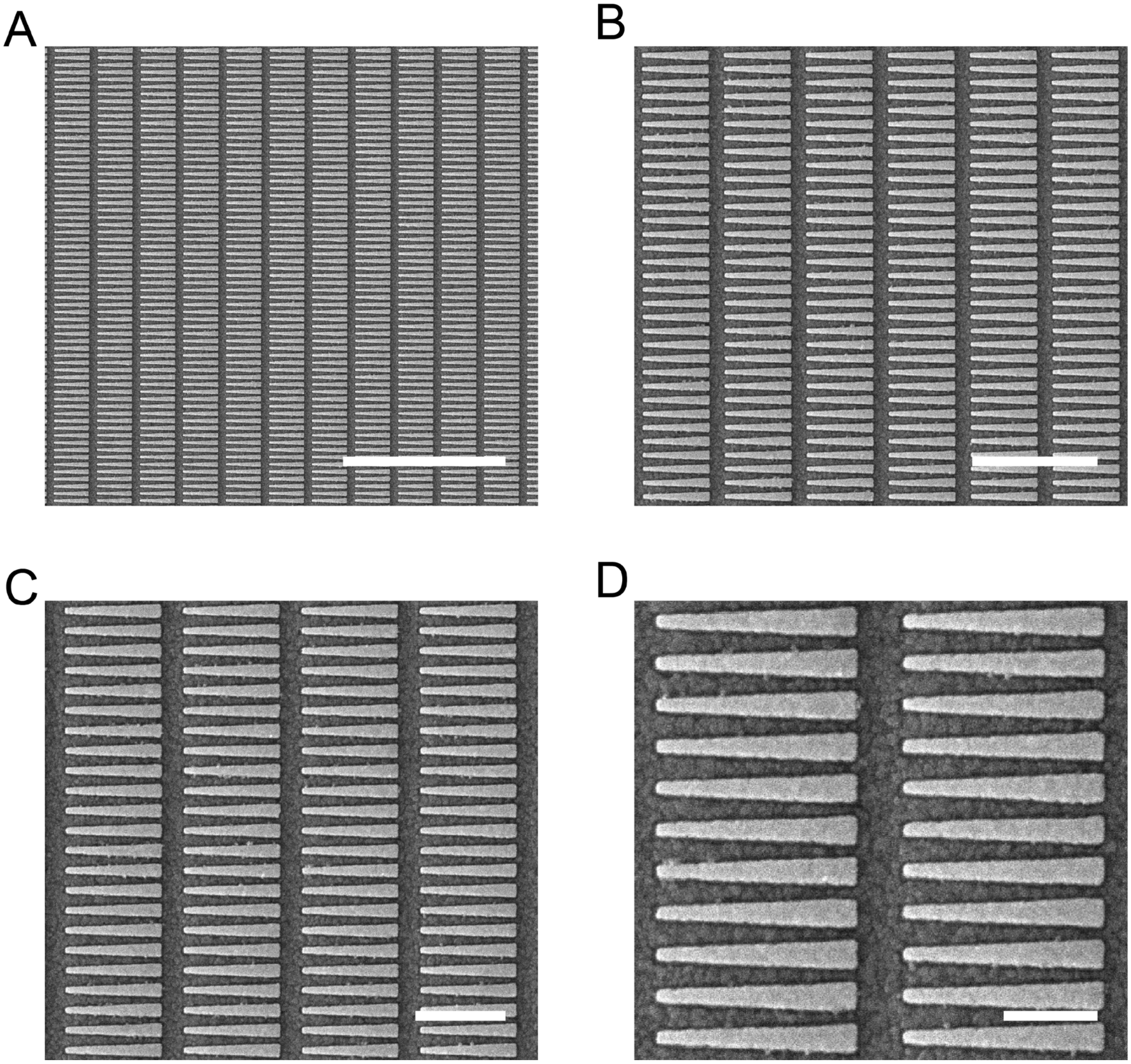

Figure S21. SEM images of the metasurface after Cu electrodeposition at various magnifications.

(A) Scale bar: 5 µm. (B) Scale bar: 2 µm. (C) Scale bar: 1 µm. (D) Scale bar: 500 nm.

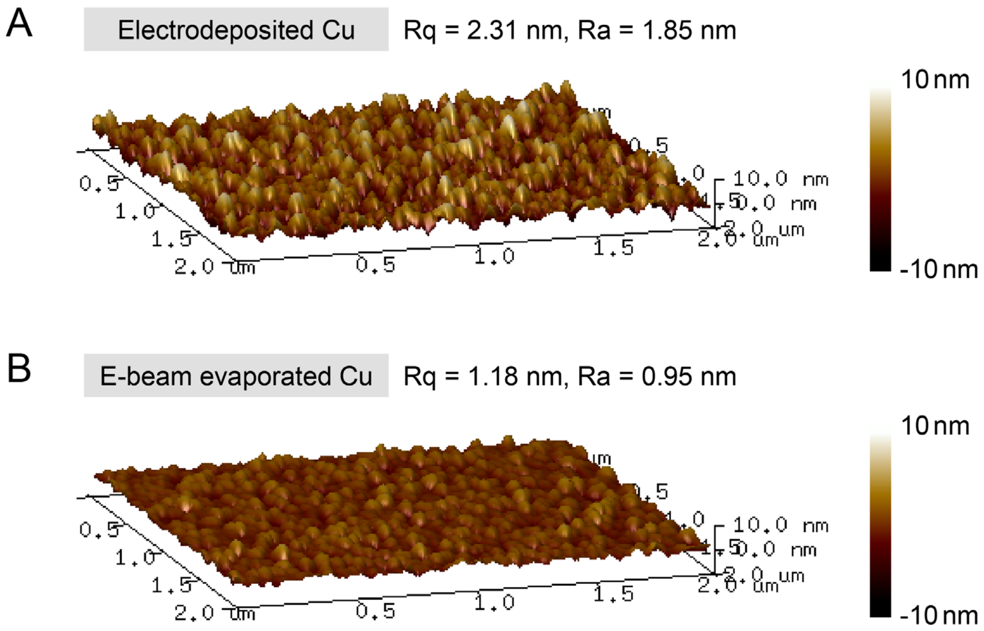

Figure S22. AFM characterization of Cu surfaces prepared by electrodeposition (A) and e-beam evaporation (B). In both cases, Cu (nominally 30 nm thick) was deposited on a silicon wafer coated with a 150 nm Au layer. For electrodeposition, a potential of -1 V was applied until the target thickness was reached, using an electrolyte containing 1 M $Cu(ClO_4)_2$, 1 M $LiClO_4$, and 20 mM $HClO_4$. For e-beam evaporation, the deposition rate was maintained at 1 Å/s.

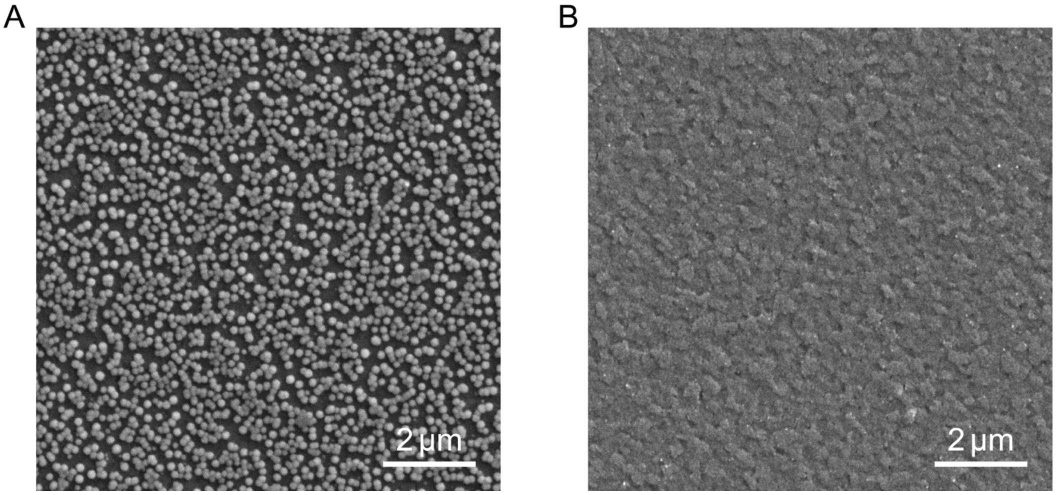

Figure S23. SEM images of the electrodeposited Cu on ITO glass electrodes before (A) and after (B) modification with an ultrathin Pt film. The commercial ITO-coated glasses (MSE Supplies LLC) have a sheet resistance of 9-15 Ω/sq, as provided by the manufacturer. Both samples were electrodeposited under identical conditions with charge density of 81.3 mC/cm^2, corresponding to a nominal Cu thickness of 30 nm assuming a uniformly flat film. The results show that electrodeposition of Cu on the transparent ITO electrode forms isolated particles rather than a continuous film, whereas surface modification with an ultrathin Pt layer promotes the formation of a more uniform and continuous Cu film.

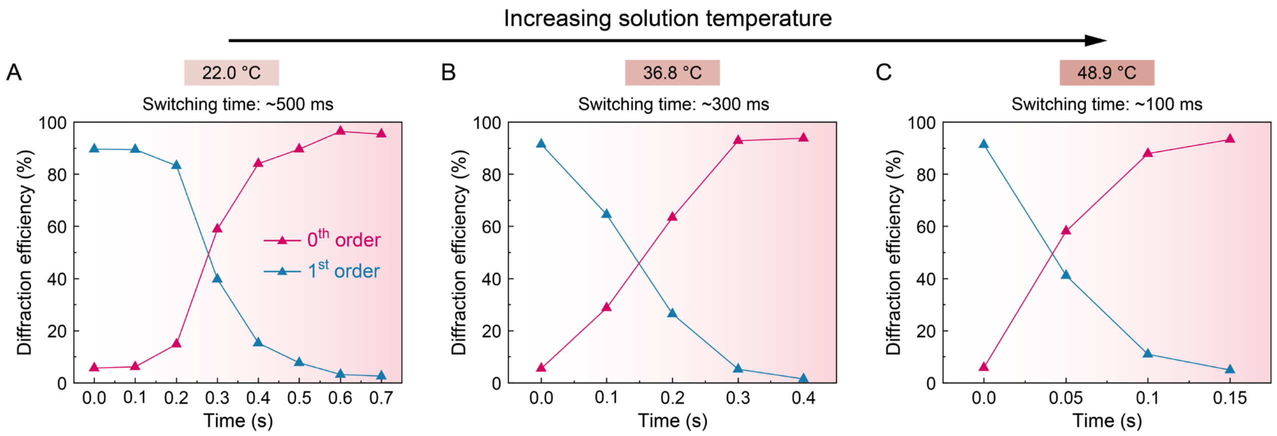

Figure S24. Electrodeposition speed characterization at various temperatures of 22.0 °C (A), 36.8 °C (B), and 48.9 °C (C). Experiments were conducted with the electrochemical cell heated via a hot plate, with the solution temperature monitored by a thermocouple immersed in the electrolyte. All electrodepositions were conducted under an applied potential of -1 V in an electrolyte containing 1 M $Cu(ClO_4)_2$, 1 M $LiClO_4$, and 20 mM $HClO_4$. The results reveal that increasing the solution temperature effectively accelerates the electrodeposition process. As the electrochemical reaction speed rate is temperature-dependent, thermal conditions must be carefully considered to ensure stable dynamic switching for real-world applications. Such thermal influences may arise from optical heating during laser illumination or from environmental temperature fluctuations. To maintain reliable switching performance, a calibrated control algorithm that dynamically adjusts the electrical parameters applied to the electrochemical system, such as voltage, can be used to compensate for temperature variations. Additionally, implementing a thermal management system in the electronic setup can also be helpful. These results also suggest that intentional heating offers a promising strategy for substantially increasing the switching speed of RME devices.

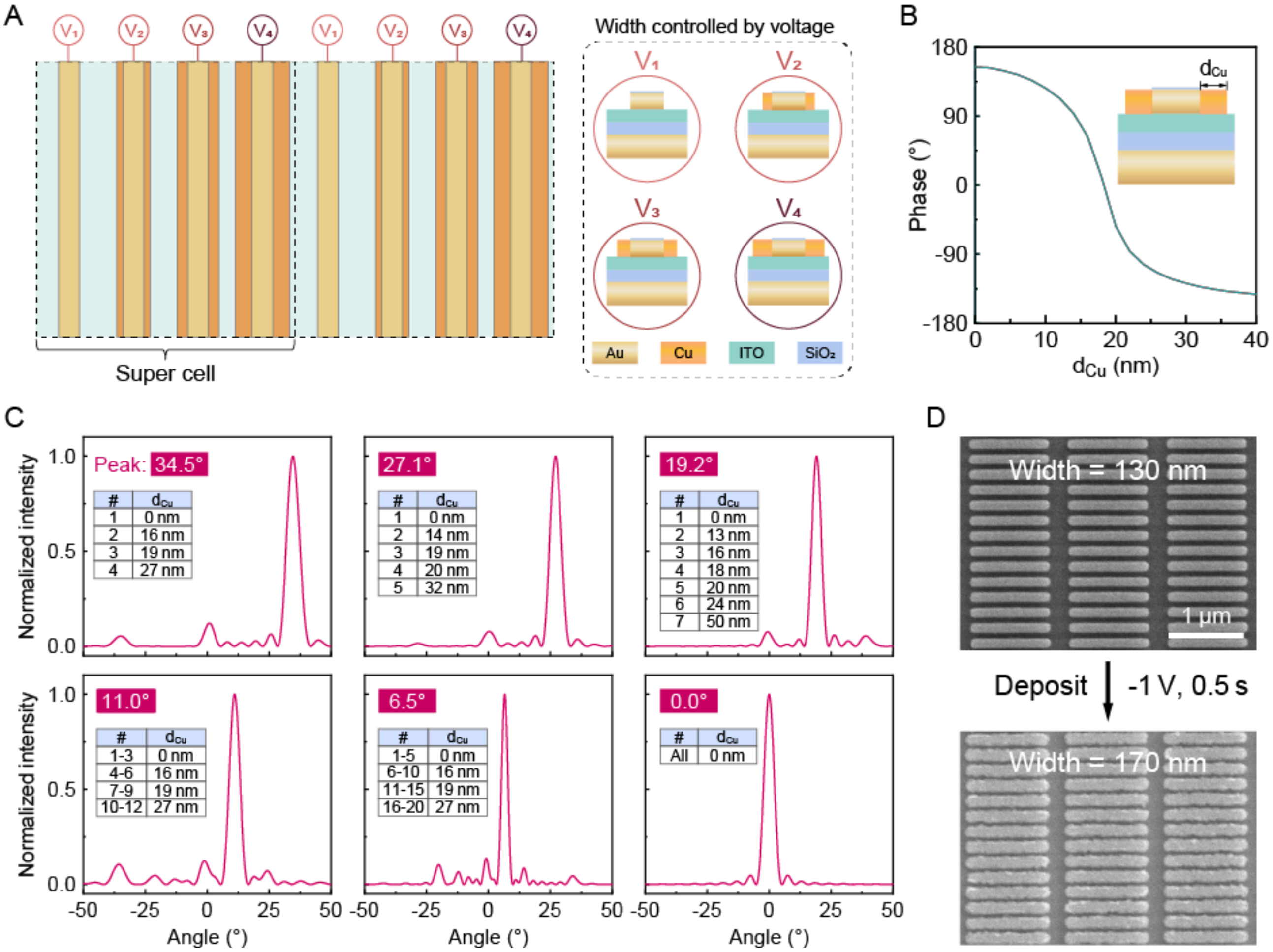

Figure S25. Design and potential of continuous-angle beam steering. (A) Metasurface with grating-shaped MIM resonators, where the grating width is individually tunable via RME with different applied voltages. Note that the top area of Au gratings is electrically insulated by an ultrathin SiO_2 film to avoid Cu electrodeposition. (B) Reflection phase as a function of lateral Cu deposition width. The intermediate ITO and SiO2 films are both 15 nm thick, and the Au gratings are 30 nm thick. (C) Simulated far-field intensity profiles of reflected light with various steering angles, controlled by the super cell pattern of gratings. Insets list the corresponding super cell pattern parameters. For the simulations, the light is normally incident with a wavelength of 685 nm. (D) Top-view SEM images of fabricated metasurface with tunable antenna width via RME of Cu. The electrodeposition was operated with an electrolyte containing 1 M $Cu(ClO_4)_2$, 1 M $LiClO_4$, and 20 mM $HClO_4$.

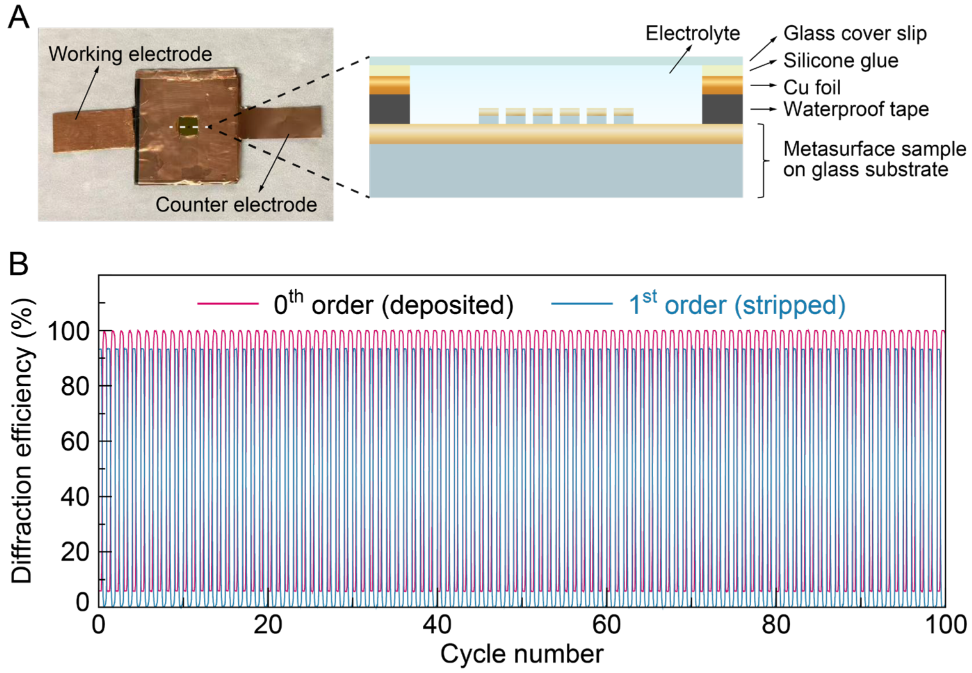

Figure S26. Demonstration of a fabricated device with RME-based dynamic metasurface for beam steering. (A) Photograph and cross-section schematic of the fabricated device. (B) Operational characterization of dynamic beam steering in terms of the 0th- and 1st-order diffraction efficiencies at the deposited and stripped states, for 100 cycles. The cycling was performed by applying -0.8 V for 5 s during deposition and 0.6 V for 10 s during stripping in an electrolyte composed of 0.1 M $Cu(ClO_4)_2$, 0.1 M $LiClO_4$, and 2 mM $HClO_4$.The results further show a highly stable and reversible switching behavior of the RME-based metasurface, as demonstrated in the in-situ and ex-situ experiments in the main text (Fig. 4).

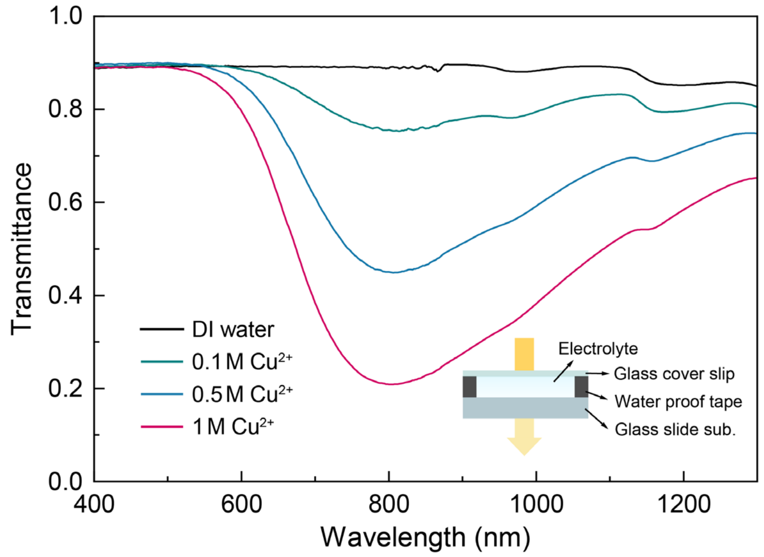

Figure S27. Measured transmittance spectra of electrolytes with varying Cu^{2+} concentrations. The measurement cells have a similar architecture to that of the electrochemical cell in Fig. S26, designed to assess electrolyte absorption under comparable thickness (~1 mm). Three different electrolyte compositions were used: (1) 1 M $Cu(ClO4)_2$, 1 M $LiClO_4$, and 20 mM $HClO_4$; (2) 0.5 M $Cu(ClO4)_2$, 0.5 M $LiClO_4$, and 10 mM $HClO_4$; (3) 0.1 M $Cu(ClO4)_2$, 0.1 M $LiClO_4$, and 2 mM $HClO_4$.

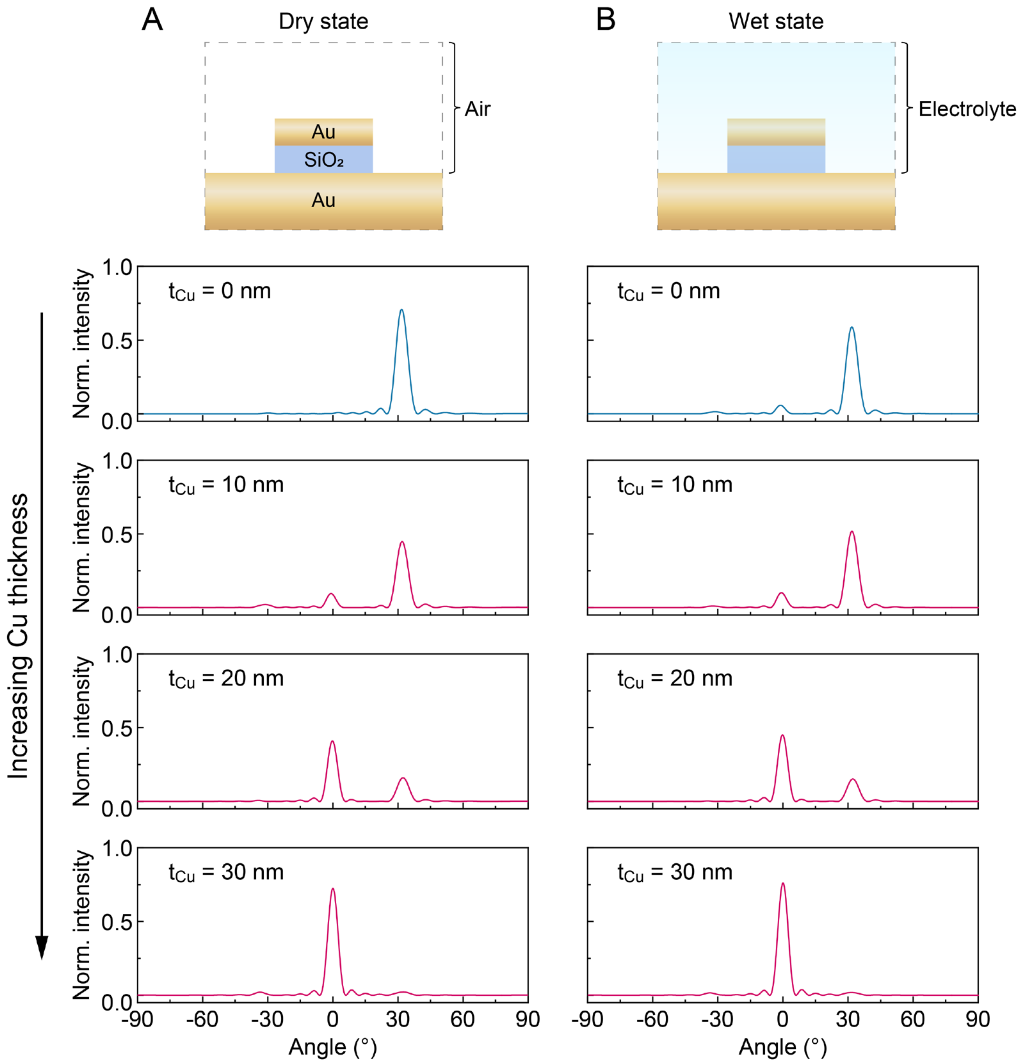

Figure S28. Simulated far-field intensity profiles with increasing Cu deposition thickness for the dry- (A) and wet-state (B) devices. For the wet-state simulations, the electrolyte is 500 nm thick. The results are normalized to the peak reflection intensity of an Ag mirror. The geometric parameters not indicated in the schematics are identical to those in Fig. 3 in the main text. The light is normally incident with a wavelength of 685 nm. Optical properties of the electrolyte are assumed to be those of water (*18*). The results support the observation that both dry-state (without electrolyte, Fig. 4E) and wet-state (with electrolyte, Fig. 4D) measurements exhibit similarly high diffraction efficiencies exceeding 90%.

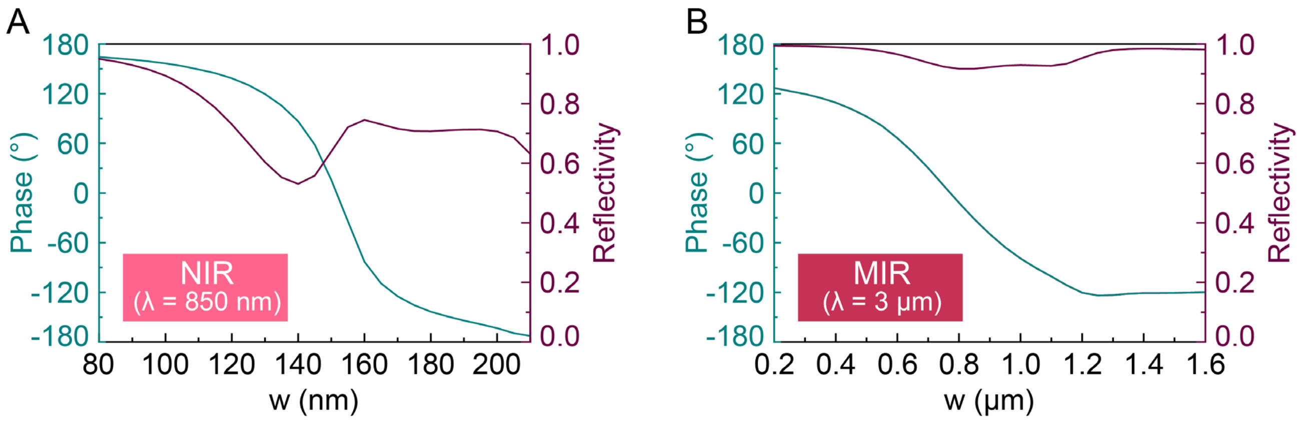

Figure S29. Simulated phase and reflectivity profiles of the near-IR (A) and mid-IR (B) designs for realizing anomalous reflection. The structural parameters, except the varying antenna width, are identical to those used in Figs. S12 and S13 for the near- and mid-IR designs, respectively.

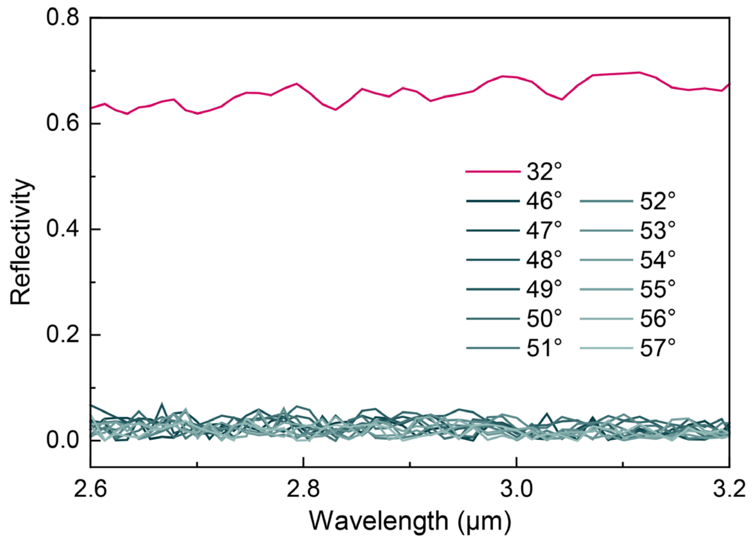

Figure S30. Measured reflectivity spectra at various detection angles for the mid-IR metasurface after Cu electrodeposition. The results show that no noticeable scattering signal was observed over a wide range of detection angles in the deposited state, indicating minimal surface-roughness-induced scattering from the electrodeposited Cu layer in the mid-IR regime.

Table S1. Optical properties of different materials for the reflectivity calculations

Regime	Material	State	Wavelength	*n*	*k*	Ref.
VIS	GST-225	Amorphous	600 nm	3.74	1.64	(*19*)
		Crystalline		3.73	3.80	
	Sb_2S_3	Amorphous		2.92	0.11	(*20*)
		Crystalline		3.83	0.57	
	Sb_2Se_3	Amorphous		4.05	0.72	(*21*)
		Crystalline		5.09	1.69	
	PANI	Oxidized (0.5 V_{SCE})		1.16	0.19	(*22*)
		Reduced (-0.2 V_{SCE})		1.64	0.00	
	VO_2	Insulating		2.85	0.37	(*23*)
		Metallic		1.67	0.77	
	Zn(s) / Zn^{2+}(aq)	Zn^{2+}(aq)		1.33	0.00	(*18*)
		Zn(s)		13.58	2.85	(*24*)
	Cu(s) / Cu^{2+}(aq)	Cu^{2+}(aq)		1.33	0.00	(*18*)
		Cu(s)		0.55	3.35	(*14*)
	Ag(s) / Ag^{+}(aq)	Ag^{+}(aq)		1.33	0.00	(*18*)
		Ag(s)		0.06	3.89	(*15*)
NIR	PANI	Oxidized (0.5 V_{SCE})	820 nm	0.99	0.82	(*22*)
		Reduced (-0.2 V_{SCE})		1.63	0.02	
	GST-225	Amorphous	1500 nm	3.91	0.05	(*19*)
		Crystalline		6.66	1.21	
	PEDOT:PSS	Insulating		1.38	0.00	(*25*)
		Metallic		0.45	0.82	
	VO_2	Insulating		3.07	0.35	(*23*)
		Metallic		1.55	1.70	
	Zn(s) / Zn^{2+}(aq)	Zn^{2+}(aq)		1.32	0.00	(*18*)
		Zn(s)		3.80	12.44	(*24*)
	Cu(s) / Cu^{2+}(aq)	Cu^{2+}(aq)		1.32	0.00	(*18*)
		Cu(s)		0.74	10.02	(*14*)
	Ag(s) / Ag^{+}(aq)	Ag^{+}(aq)		1.32	0.00	(*18*)
		Ag(s)		0.24	10.54	(*15*)
	ITO	0 V	1550 nm	1.29	0.15	(*26*)
		3.5 V (Gating)		0.39	0.94	

	InSb	Amorphous	3333 nm	4.98	0.03	(*27*)
		Crystalline		3.89	0.10	
	GST-225	Amorphous		4.17	0.00	(*28*)
		Crystalline		6.45	1.91	
	GSS1T4	Amorphous		4.22	0.00	
		Crystalline		6.44	1.22	
	GSS3T2	Amorphous		3.87	0.00	
		Crystalline		5.80	0.79	
MIR	VO_2	Insulating	5000 nm	3.19	0.02	(*29*)
		Metallic		3.37	4.11	
	Zn(s) / Zn^{2+}(aq)	Zn^{2+}(aq)		1.33	0.01	(*18*)
		Zn(s)		17.87	51.35	(*24*)
	Cu(s) / Cu^{2+}(aq)	Cu^{2+}(aq)		1.33	0.01	(*18*)
		Cu(s)		3.26	33.00	(*14*)
	Ag(s) / Ag^{+}(aq)	Ag^{+}(aq)		1.33	0.01	(*18*)
		Ag(s)		2.60	35.31	(*15*)

Table S2. Up-to-date active beam-steering metasurfaces

Operating regime	Active material	Metasurface type	Diffraction efficiency	SNR	Ref.
VIS/NIR/MIR	RME of Cu	MIM resonator	>90%	>12 dB	This work
VIS	Liquid crystal	Grating	50%	0.3 dB (504 nm)	(*30*)
	Liquid crystal	Dielectric disk	57%	3.2 dB (660 nm)	(*31*)
	Liquid crystal	Dielectric disk	50%	0.5 dB (745 nm)	(*32*)
	PEDOT	Geometric phase	4%	-14.4 dB (750 nm)	(*33*)
	Water immersion/ drying	Gradient effective index	71%	3.8 dB (475 nm)	(*34*)
	WS_2	Grating	NA	NA (620 nm)	(*35*)
	WS_2	Grating	0.2%	-26.8 dB (636 nm)	(*36*)
NIR	ITO	Grating	48%	2.7 dB (1340 nm)	(*37*)
	ITO	Dielectric disk	4%	-13.6 dB (1470 nm)	(*38*)
	ITO	Grating	70%	4.1 dB (1510 nm)	(*39*)
	ITO	Grating	74%	5.8 dB (1515 nm)	(*40*)
	ITO	Grating	NA	-1.9 dB (1550 nm)	(*26*)
	GSST	Dielectric disk	56%	2.4 dB (1550 nm)	(*41*)
	GST-225	Geometric phase	39%	-0.4 dB (1600 nm)	(*42*)
	PANI	Dielectric disk	71%	4.0 dB (785 nm)	(*43*)
	Si	Grating	13%	-5.3 dB (1550 nm)	(*44*)
	Multiple quantum wells	Grating	14%	-7.8 dB (917 nm)	(*45*)
MIR	PEDOT:PSS	Geometric phase	50%	0 dB (2650 nm)	(*25*)
	GST-326	Geometric phase	16%	-7.3 dB (3100 nm)	(*46*)
	Graphene	Metal resonator	NA	NA (8600 nm)	(*47*)

Legends for Supplementary Movies

Movie S1. In-situ beam steering characterization for 1-10 cycles (video speed ×10)

Movie S2. In-situ beam steering characterization for 591-600 cycles (video speed ×10)

References

1. J. N. Fuller, Thomas F., Harb, Electrochemical Engineering. John Wiley & Sons Ltd (2018).
2. T. I. Quickenden, Q. Xu, Toward a Reliable Value for the Diffusion Coefficient of Cupric Ion in Aqueous Solution. *J. Electrochem. Soc.* **143**, 1248 (1996).
3. N. Jeon, J. Noh, C. Jung, J. Rho, Electrically tunable metasurfaces: from direct to indirect mechanisms. *New J. Phys.* **24**, 075001 (2022).
4. D. T. Schoen, A. L. Holsteen, M. L. Brongersma, Probing the electrical switching of a memristive optical antenna by STEM EELS. *Nat. Commun.* **7**, 12162 (2016).
5. C. J. Barile, D. J. Slotcavage, J. Hou, M. T. Strand, T. S. Hernandez, M. D. McGehee, Dynamic Windows with Neutral Color, High Contrast, and Excellent Durability Using Reversible Metal Electrodeposition. *Joule* **1**, 133–145 (2017).
6. C. Sui, J. Pu, T.-H. Chen, J. Liang, Y.-T. Lai, Y. Rao, R. Wu, Y. Han, K. Wang, X. Li, V. Viswanathan, P.-C. Hsu, Dynamic electrochromism for all-season radiative thermoregulation. *Nat. Sustain.* **6**, 428–437 (2023).
7. T. S. Hernandez, M. Alshurafa, M. T. Strand, A. L. Yeang, M. G. Danner, C. J. Barile, M. D. McGehee, Electrolyte for Improved Durability of Dynamic Windows Based on Reversible Metal Electrodeposition. *Joule* **4**, 1501–1513 (2020).
8. K. Yan, Z. Lu, H.-W. Lee, F. Xiong, P.-C. Hsu, Y. Li, J. Zhao, S. Chu, Y. Cui, Selective deposition and stable encapsulation of lithium through heterogeneous seeded growth. *Nat. Energy* **1**, 16010 (2016).
9. Y. Rao, J. Dai, C. Sui, Y.-T. Lai, Z. Li, H. Fang, X. Li, W. Li, P.-C. Hsu, Ultra-Wideband Transparent Conductive Electrode for Electrochromic Synergistic Solar and Radiative Heat Management. *ACS Energy Lett.* **6**, 3906–3915 (2021).
10. M. Li, D. Liu, H. Cheng, L. Peng, M. Zu, Manipulating metals for adaptive thermal camouflage. *Sci. Adv.* **6**, eaba3494 (2020).
11. A. B. Vasista, D. K. Sharma, G. V. P. Kumar, Fourier plane optical microscopy and spectroscopy. In Encyclopedia of Applied Physics (ed. G. L. Trigg). Weinheim: Wiley-VCH (2019).

12. J. Kischkat, S. Peters, B. Gruska, M. Semtsiv, M. Chashnikova, M. Klinkmüller, O. Fedosenko, S. Machulik, A. Aleksandrova, G. Monastyrskyi, Y. Flores, W. Ted Masselink, Mid-infrared optical properties of thin films of aluminum oxide, titanium dioxide, silicon dioxide, aluminum nitride, and silicon nitride. *Appl. Opt.* **51**, 6789–6798 (2012).
13. S. Babar, J. H. Weaver, Optical constants of Cu, Ag, and Au revisited. *Appl. Opt.* **54**, 477–481 (2015).
14. M. A. Ordal, R. J. Bell, R. W. Alexander, L. L. Long, M. R. Querry, Optical properties of fourteen metals in the infrared and far infrared: Al, Co, Cu, Au, Fe, Pb, Mo, Ni, Pd, Pt, Ag, Ti, V, and W. *Appl. Opt.* **24**, 4493–4499 (1985).
15. H. U. Yang, J. D'Archangel, M. L. Sundheimer, E. Tucker, G. D. Boreman, M. B. Raschke, Optical dielectric function of silver. *Phys. Rev. B* **91**, 235137 (2015).
16. A. G. Mathewson, H. P. Myers, Absolute Values of the Optical Constants of Some Pure Metals. *Phys. Scr.* **4**, 291 (1971).
17. K. M. McPeak, S. V Jayanti, S. J. P. Kress, S. Meyer, S. Iotti, A. Rossinelli, D. J. Norris, Plasmonic Films Can Easily Be Better: Rules and Recipes. *ACS Photonics* **2**, 326–333 (2015).
18. G. M. Hale, M. R. Querry, Optical Constants of Water in the 200-nm to 200-μm Wavelength Region. *Appl. Opt.* **12**, 555–563 (1973).
19. J. Zheng, A. Khanolkar, P. Xu, S. Colburn, S. Deshmukh, J. Myers, J. Frantz, E. Pop, J. Hendrickson, J. Doylend, N. Boechler, A. Majumdar, GST-on-silicon hybrid nanophotonic integrated circuits: a non-volatile quasi-continuously reprogrammable platform. *Opt. Mater. Express* **8**, 1551–1561 (2018).
20. H. Liu, W. Dong, H. Wang, L. Lu, Q. Ruan, Y. S. Tan, R. E. Simpson, J. K. W. Yang, Rewritable color nanoprints in antimony trisulfide films. *Sci. Adv.* **6**, eabb7171 (2024).
21. M. Delaney, I. Zeimpekis, D. Lawson, D. W. Hewak, O. L. Muskens, A New Family of Ultralow Loss Reversible Phase-Change Materials for Photonic Integrated Circuits: Sb2S3 and Sb2Se3. *Adv. Funct. Mater.* **30**, 2002447 (2020).
22. C. Barbero, R. Kötz, Nanoscale Dimensional Changes and Optical Properties of Polyaniline Measured by In Situ Spectroscopic Ellipsometry. *J. Electrochem. Soc.* **141**, 859 (1994).

23. I. Derkaoui, M. Khenfouch, M. Benkhali, A. Rezzouk, VO2 thin films for smart windows: Numerical study of the optical properties and performance improvement. *J. Phys. Conf. Ser.* **1292**, 12010 (2019).

24. M. R. Querry, Optical constants of minerals and other materials from the millimeter to the ultraviolet. U.S. Army Report CRDEC-CR-88009 (1987).

25. J. Karst, M. Floess, M. Ubl, C. Dingler, C. Malacrida, T. Steinle, S. Ludwigs, M. Hentschel, H. Giessen, Electrically switchable metallic polymer nanoantennas. *Science* **374**, 612–616 (2021).

26. Y.-W. Huang, H. W. H. Lee, R. Sokhoyan, R. A. Pala, K. Thyagarajan, S. Han, D. P. Tsai, H. A. Atwater, Gate-Tunable Conducting Oxide Metasurfaces. *Nano Lett.* **16**, 5319–5325 (2016).

27. A.-K. U. Michel, D. N. Chigrin, T. W. W. Maß, K. Schönauer, M. Salinga, M. Wuttig, T. Taubner, Using Low-Loss Phase-Change Materials for Mid-Infrared Antenna Resonance Tuning. *Nano Lett.* **13**, 3470–3475 (2013).

28. Y. Zhang, J. B. Chou, J. Li, H. Li, Q. Du, A. Yadav, S. Zhou, M. Y. Shalaginov, Z. Fang, H. Zhong, C. Roberts, P. Robinson, B. Bohlin, C. Ríos, H. Lin, M. Kang, T. Gu, J. Warner, V. Liberman, K. Richardson, J. Hu, Broadband transparent optical phase change materials for high-performance nonvolatile photonics. *Nat. Commun.* **10**, 4279 (2019).

29. J. Barker, H. Verleur, H. Guggenheim, Infrared optical properties of vanadium dioxide above and below the transition temperature. *Phys. Rev. Lett.* **17**, 1286–1289 (1966).

30. S. Mansha, P. Moitra, X. Xu, T. W. W. Mass, R. M. Veetil, X. Liang, S. Q. Li, R. Paniagua-Domínguez, A. I. Kuznetsov, High resolution multispectral spatial light modulators based on tunable Fabry-Perot nanocavities. *Light Sci. Appl.* **11** (2022).

31. S.-Q. Li, X. Xu, R. Maruthiyodan Veetil, V. Valuckas, R. Paniagua-Domínguez, A. I. Kuznetsov, Phase-only transmissive spatial light modulator based on tunable dielectric metasurface. *Science* **364**, 1087–1090 (2019).

32. A. Komar, R. Paniagua-Domínguez, A. Miroshnichenko, Y. F. Yu, Y. S. Kivshar, A. I. Kuznetsov, D. Neshev, Dynamic Beam Switching by Liquid Crystal Tunable Dielectric Metasurfaces. *ACS Photonics* **5**, 1742–1748 (2018).

33. J. Ratzsch, J. Karst, J. Fu, M. Ubl, T. Pohl, F. Sterl, C. Malacrida, M. Wieland, B. Reineke, T.

Zentgraf, S. Ludwigs, M. Hentschel, H. Giessen, Electrically switchable metasurface for beam steering using PEDOT polymers. *J. Opt.* **22**, 124001 (2020).

34. Z. Li, C. Wan, C. Dai, J. Zhang, G. Zheng, Z. Li, Actively Switchable Beam-Steering via Hydrophilic/Hydrophobic-Selective Design of Water-Immersed Metasurface. *Adv. Opt. Mater.* **9**, 2100297 (2021).

35. Q. Li, J.-H. Song, F. Xu, J. van de Groep, J. Hong, A. Daus, Y. J. Lee, A. C. Johnson, E. Pop, F. Liu, M. L. Brongersma, A Purcell-enabled monolayer semiconductor free-space optical modulator. *Nat. Photonics* **17**, 897–903 (2023).

36. L. Guarneri, T. Bauer, Q. Li, J.-H. Song, S. Selvin, A. P. Saunders, F. Liu, M. L. Brongersma, J. van de Groep, Dynamic Excitonic Beam Switching with Atomically-Thin Binary Blazed Gratings. *Adv. Opt. Mater.* **13**, 2403257 (2025).

37. J. Park, B. G. Jeong, S. Il Kim, D. Lee, J. Kim, C. Shin, C. B. Lee, T. Otsuka, J. Kyoung, S. Kim, K.-Y. Yang, Y.-Y. Park, J. Lee, I. Hwang, J. Jang, S. H. Song, M. L. Brongersma, K. Ha, S.-W. Hwang, H. Choo, B. L. Choi, All-solid-state spatial light modulator with independent phase and amplitude control for three-dimensional LiDAR applications. *Nat. Nanotechnol.* **16**, 69–76 (2021).

38. A. Howes, W. Wang, I. Kravchenko, J. Valentine, Dynamic transmission control based on all-dielectric Huygens metasurfaces. *Optica* **5**, 787–792 (2018).

39. G. K. Shirmanesh, R. Sokhoyan, P. C. Wu, H. A. Atwater, Electro-optically Tunable Multifunctional Metasurfaces. *ACS Nano* **14**, 6912–6920 (2020).

40. C. R. de Galarreta, A. M. Alexeev, Y.-Y. Au, M. Lopez-Garcia, M. Klemm, M. Cryan, J. Bertolotti, C. D. Wright, Nonvolatile Reconfigurable Phase-Change Metadevices for Beam Steering in the Near Infrared. *Adv. Funct. Mater.* **28**, 1704993 (2018).

41. Y. Zhang, C. Fowler, J. Liang, B. Azhar, M. Y. Shalaginov, S. Deckoff-Jones, S. An, J. B. Chou, C. M. Roberts, V. Liberman, M. Kang, C. Ríos, K. A. Richardson, C. Rivero-Baleine, T. Gu, H. Zhang, J. Hu, Electrically reconfigurable non-volatile metasurface using low-loss optical phase-change material. *Nat. Nanotechnol.* **16**, 661–666 (2021).

42. C. Choi, S.-Y. Lee, S.-E. Mun, G.-Y. Lee, J. Sung, H. Yun, J.-H. Yang, H.-O. Kim, C.-Y.

Hwang, B. Lee, Metasurface with Nanostructured Ge2Sb2Te5 as a Platform for Broadband-Operating Wavefront Switch. *Adv. Opt. Mater.* **7**, 1900171 (2019).

43. W. Lu, L. de S. Menezes, A. Tittl, H. Ren, S. A. Maier, Active Huygens' metasurface based on in-situ grown conductive polymer. *Nanophotonics* **13**, 39–49 (2024).

44. Y. Horie, A. Arbabi, E. Arbabi, S. M. Kamali, A. Faraon, High-Speed, Phase-Dominant Spatial Light Modulation with Silicon-Based Active Resonant Antennas. *ACS Photonics* **5**, 1711–1717 (2018).

45. P. C. Wu, R. A. Pala, G. Kafaie Shirmanesh, W.-H. Cheng, R. Sokhoyan, M. Grajower, M. Z. Alam, D. Lee, H. A. Atwater, Dynamic beam steering with all-dielectric electro-optic III–V multiple-quantum-well metasurfaces. *Nat. Commun.* **10**, 3654 (2019).

46. X. Yin, T. Steinle, L. Huang, T. Taubner, M. Wuttig, T. Zentgraf, H. Giessen, Beam switching and bifocal zoom lensing using active plasmonic metasurfaces. *Light Sci. Appl.* **6**, e17016–e17016 (2017).

47. M. C. Sherrott, P. W. C. Hon, K. T. Fountaine, J. C. Garcia, S. M. Ponti, V. W. Brar, L. A. Sweatlock, H. A. Atwater, Experimental Demonstration of >230° Phase Modulation in Gate-Tunable Graphene–Gold Reconfigurable Mid-Infrared Metasurfaces. *Nano Lett.* **17**, 3027–3034 (2017).
